# Paramedic Practitioners within ambulance services: views of Australian policymakers, health professionals, and consumers

**DOI:** 10.1186/s12913-025-12614-y

**Published:** 2025-04-10

**Authors:** Matt Wilkinson-Stokes, Celene Y. L. Yap, Di Crellin, Ray Bange, George Braitberg, Marie Gerdtz

**Affiliations:** 1 Faculty of Medicine, Dentistry, and Health Sciences, The University of Melbourne, Grattan Street, Parkville, VIC 3054 Australia; 2 School of Health, Medical, and Applied Sciences, Central Queensland University, Bruce Highway, Rockhampton, QLD 4702 Australia; 3 School of Health, University of the Sunshine Coast, Sippy Downs Drive, Sippy Downs, QLD 4556 Australia; 4 Department of Emergency Medicine, Austin Health, Studley Road, Heidelberg, VIC 3084 Australia; 5 School of Nursing and Midwifery, La Trobe University, Plenty Road, Bundoora, VIC 3086 Australia

**Keywords:** Ambulance services, Community paramedicine, Emergency medical service, Out-of-hospital, Paramedic Practitioner, Primary health care

## Abstract

**Background:**

Globally, ambulance services face overwhelming primary and urgent care presentations that they are not structurally or culturally designed to manage efficiently or effectively. One mechanism to meet this consumer demand is the implementation of Paramedic Practitioner models with postgraduate qualifications in primary and urgent care. This study explores interest-holder views on reactive Paramedic Practitioner models within Australian ambulance services.

**Methods:**

A multidisciplinary team representing ambulance services was formed, including paramedicine, nursing, and medicine. A realist lens was adopted, and a qualitative research design using inductive thematic analysis employed. Semi-structured focus groups or interviews were conducted to obtain data from 56 participants. Interest-holders represented included consumers (*n* = 16), members of parliament (*n* = 3), government executives (*n* = 8), industry executives representing emergency medicine, general practice, nursing, and paramedicine (*n* = 6), ambulance service executives and medical directors (*n* = 7), researchers (*n* = 8), and practicing clinicians from paramedicine, nursing, and medicine (*n* = 8).

**Results:**

Consumers described calling ambulance services for non-emergency complaints as they didn’t know if their concern was an emergency or not, not wanting to go to hospital, and wanting someone to listen to them, reassure them, and then quickly solve their problem on the spot: they saw Paramedic Practitioners as aptly meeting this need. Among the healthcare professions, opinions were divided. Most participants were largely unfamiliar with Paramedic Practitioners or the evidence base supporting this model of practice, the concept received widespread support at the clinician level, and a small but avidly dissenting contingent of national policymakers opposed the models. Paramedic Practitioner models were considered to require broad support across the healthcare system to be effective. Policymakers were unsure which outcomes they wanted measured to evaluate models.

**Conclusion:**

This study reports a wide range of interest-holder perspectives on the use of reactive Paramedic Practitioners within Australian ambulance services. Enablers (*n* = 10) and barriers (*n* = 10) to efficient and effective Paramedic Practitioner models were identified. Key outcomes of interest (*n* = 6) were identified, and these may be operationalised in future evaluations of reactive Paramedic Practitioner programs.

**Supplementary Information:**

The online version contains supplementary material available at 10.1186/s12913-025-12614-y.

## Background

Government-sponsored ambulance services in Australia are mandated to respond to all healthcare-related ‘000’ (the Australian emergency telephone number) requests for service [1]. While historically their response was exclusively transport, ambulance services have evolved with medical evidence and societal expectations to include treatment (for example, resuscitation), and have since adopted a continually expanding scope of practice [2, 3]. Ambulance service responses in Australia most commonly include both an ambulance vehicle and two degree-qualified paramedics.

Approximately 60% of ‘000’ requests to ambulance services in Australia are not classified as healthcare emergencies, with urgent and primary healthcare presentations now dominating workload [4–7]. This has previously been raised in government reports in New South Wales in 2011, Tasmania in 2017, Australian Capital Territory in 2018, Queensland in 2022, and nationally in a 2022 Grattan Institute report [8–12]. Beyond Australia, this has been discussed in at least 11 academic reviews, and Oxford University currently host a website stating that ambulance service paramedics 'see the same types of patients as GPs' (General Practitioners) [6, 13–23].

One mechanism to address these consumer presentations are paramedics with expertise in primary-urgent care and working within an ambulance service to respond to non-emergency requests, referred to in this article as Paramedic Practitioners [13]. Precursors to this role were first introduced in the USA in 1992, with their initial scope of practice 33 years ago including providing courses of oral antibiotics and suturing wounds [23]. Remarkably, the model was later established completely independently in three more countries: Canada in 2001, the United Kingdom in 2002, and Australia in 2007 [13, 24–26]. By 2015 there were 48 programs in Ontario alone, and by 2017 over 150 in the United States [27]. Today, these paramedics generally complete a Master’s degree, along with further training under general practice and emergency department (ED) physicians [28]. They can work in any of ambulance services, EDs, GP clinics, urgent care centres, or autonomously – or in ‘rotational’ models across multiple practice settings (for example, one model has Paramedic Practitioners work one week in the ambulance service, followed by two weeks in two different GP clinics) [28]. They can work either reactively (e.g. responding to ‘000’ requests) or proactively (e.g. identifying high risk recent ED discharges and scheduling home visits to prevent deterioration) [28].

**Fig. 1:**
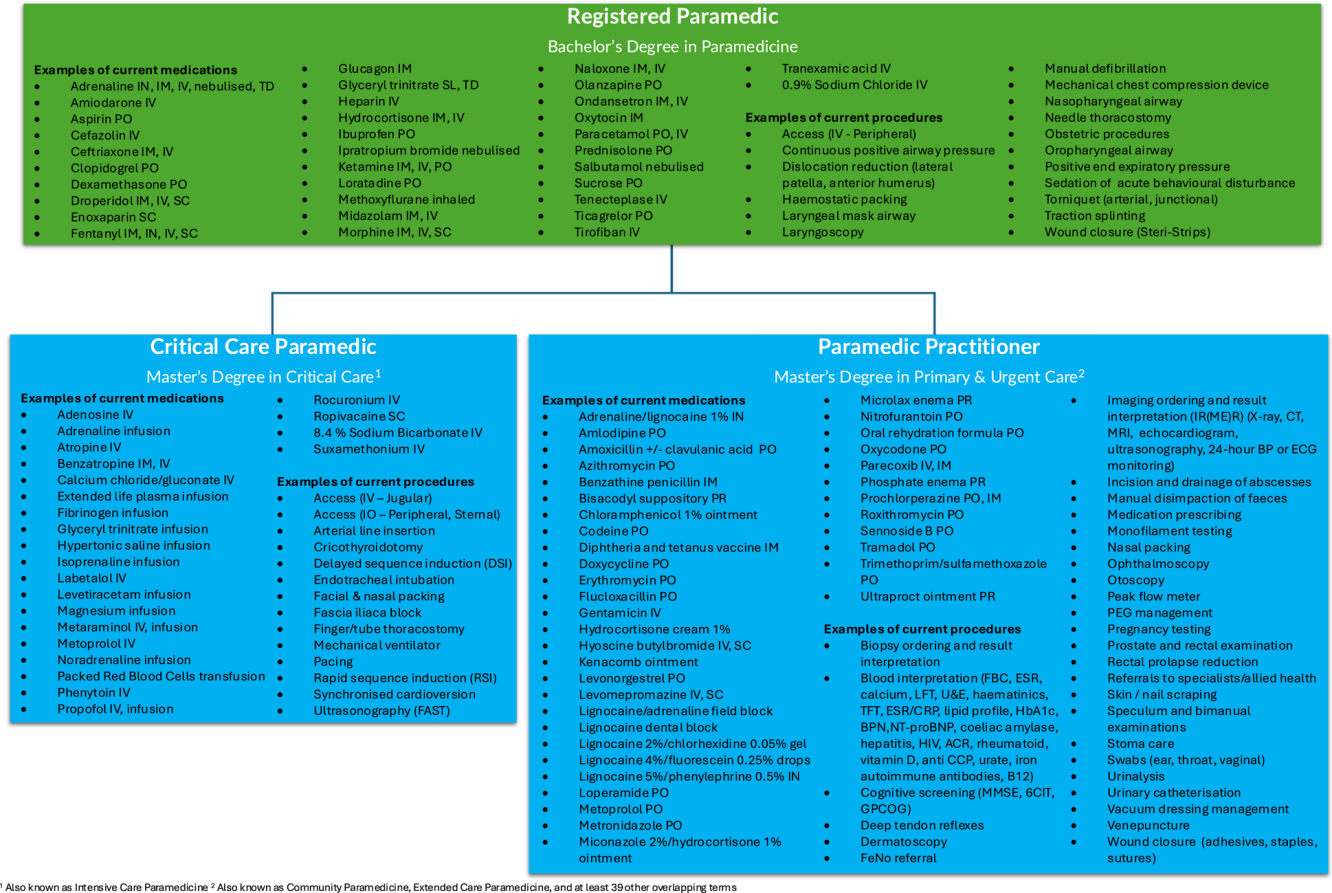
An example of current Paramedic Practitioner scope of practice, and how this relates to Registered Paramedic and Critical Care Paramedic skills. This is not a comprehensive list, and local variations are commonplace

This paper exclusively looks at one use of Paramedic Practitioners in Australia: operating reactively in an ambulance service responding to non-emergency ‘000’ requests with an expanded scope of practice [29]. An example of Paramedic Practitioner scope of practice, and how this compares to Registered Paramedic and Critical Care Paramedic (also known as Intensive Care) roles, is provided in Fig. 1.

Titles for these roles are highly inconsistent, and include Paramedic Practitioner, Extended Care Paramedic, and Community Paramedic, among 39 others found in our ongoing environmental scan [29–31]. With no consensus in terminology currently, the term Paramedic Practitioner has been used in this article as it was adopted by Parliament in our jurisdiction when legislation empowering Paramedic Practitioners with autonomous prescribing was passed this year [32]. Participant quotes included below have not been edited to use our chosen terminology, and instead use the terminology chosen by participants.

Paramedic Practitioners (or precursor roles) have existed in Australia for 18 years, now expanding to nearly every ambulance service [24, 28, 29, 33, 34]. Australian government reports have repeatedly recommended their introduction and expansion, including in 2006 in Queensland, 2014 nationally, 2017 in Tasmania, 2017 in the Northern Territory, 2018 in the Australian Capital Territory, 2019 in South Australia, 2019 in the Northern Territory, 2022 in New South Wales, 2024 in Tasmania, 2024 in New South Wales, and 2024 nationally [9, 10, 34–42]. Recently, there has been renewed focus on developing this model of care in Victoria, including a $20 million investment in a fee-free Master's degree and a recently-approved legislation to facilitate autonomous paramedic prescribing [32, 43, 44]. Additionally, in April 2024 the Health Ministers of all States and Territories agreed to implement regulatory endorsement via Ahpra, the Australian healthcare regulator; this effectively creates two levels of recognised expertise within paramedicine in Australia – Critical Care Paramedics, and Paramedic Practitioners [45]. However, as ambulance services continue to move out of their traditional emergency role and into a healthcare sub-field traditionally managed by general practices and urgent care centres, it is reasonable to expect difficulties in integration. Research on policy implementation has found that a convergence of agreement among interest-holders is necessary to facilitate change; therefore, the views of broader interest-holders beyond ambulance services will be key to the success of these programs [46–49].

While there are over 150 studies investigating various aspects of reactive Paramedic Practitioners within ambulance services specifically—and over 370 articles on Paramedic Practitioners generally, along with dozens of reports, reports, inquiry documents, and several theses – an understanding of the broader interest-holder perspectives is lacking. Such knowledge is vital to structuring this model for success, and this research directly addresses this gap.

## Methods

### Aim

This study seeks to answer the question: what are the perspectives of interest-holders on reactive Paramedic Practitioner models within Australian ambulance services?

### Design

We used a realist framework, with our method being inductive analysis of both semantic and latent themes [50, 51]. Our goal was therefore not to simply report perspectives and organise them into themes, but to go beyond this and consider what sociocultural conditions enabled these perspectives to emerge [50, 51]. Interviews and focus groups were employed to capture the nuanced details of spoken language, including latent concepts that may be implied but not explicitly stated [51–54]. Using this methodology, data expressed by participants can be organised either by how often or how intensely it is discussed, and compared by background of the participant to suggest if a particular clinical background impacts viewpoints on the topic [51–54].

Guba and Lincoln’s criteria for establishing credibility, transferability, dependability, and confirmability was considered before study commencement to maximise trustworthiness of data [55–57]. Specific methods for credibility included participants having the opportunity to review and revise their full transcript (with other participants’ quotes excluded to maintain confidentiality), then being provided with a list of specific quotations and how they were coded and allowed to modify the researcher interpretation. Transferability was ensured by outlining participant characteristics for readers. Dependability was achieved by traceable data handling – including providing codes and 18,000 words of supporting content in Appendix I – and via clear methods description. Use of the diverse multidisciplinary team and high levels of reflexivity during fortnightly team meetings aided confirmability: the team includes professors and researchers from paramedicine (RB, MWS), nursing (MG, DC, CY), and medicine (GB). To ensure adequate reporting, the Standards for Reporting Qualitative Research and Consolidated Criteria for Reporting Qualitative Research guidelines were followed [58, 59]. This study forms part of a larger environmental scan, and is the second publication from this interview series [7, 30].

### Eligibility and recruitment

To capture a wide range of viewpoints, six interest-holders categories were prospectively identified:

1. Consumer;
2. Paramedical (including Paramedic Practitioner model clinicians, directors, researchers, and educators);
3. Political;
4. Policy;
5. Medical; and,
6. Nursing.

For category 1 (consumers), open advertisement via a community organisation’s social media was used, a nominal voucher of $15 offered, and snowballing accepted, with the advertisement reproduced in Appendix II. For category 2 (Paramedic Practitioner clinicians), open advertisement in a national college’s social media was used, with no remuneration offered and snowballing accepted. For categories 2 (Paramedic Practitioner directors, researchers, and educators) and 3, specific individuals were identified by the study team and recruited directly via email. For categories 4–6, relevant organisations were contacted and asked to nominate an appropriate representative: in all cases, these were chairpersons or CEOs. A total of 56 participants were recruited.

Saturation, the point at which sampled data appropriately represents the target population, was determined using the criteria of Thorne (depth, richness, detail, and coherence) [60–65]. As analysis was conducted in tandem with recruitment, saturation was assessed continually for five months among the team while codes and themes were discussed. As this study aimed to provide a snapshot on a broad range of views, rather than to investigate any specific category of interest-holders’ views in depth, saturation was considered from the perspective of breadth across the interest-holder landscape. To meet this, the study team proposed to recruit approximately one-third each for consumers, paramedics, and all other interest-holder groups: this was considered satisfactory with 29% of the final participants being consumers, 41% paramedics, and 30% all other healthcare disciplines. The research team agreed that all saturation criteria were met, and recruitment was concluded, in August 2023.

### Interview procedures

Interviews were conducted between April and August 2023. All were audio-recorded via Zoom (Zoom Video Communications, 2022). Interviews ran for a maximum of 74 min, with a median duration of 40 min and a minimum of 27 min. Focus groups were used wherever possible due to their ability to stimulate interprofessional comparison, and a total of 16 groups including 41 participants were held, most commonly with 2–3 participants. The study team were concerned that consumers may be deferential to healthcare or political personnel on this topic, and therefore all consumer focus groups were conducted separately. Where logistical barriers precluded participants attending focus groups, individual interviews were used, and these occurred for 15 participants.

Interviews and focus groups were semi-structured, with a protocol drafted in advance, piloted in two stages, and questions revised iteratively at fortnightly meetings to investigate emerging or unresolved topics. Interview procedures are provided in full in Appendix III. To increase familiarity with the data, interviews were transcribed manually by a reviewer (MWS) using intelligent style [51, 66, 67].

### Data analysis

We used Braun and Clarke’s recursive method of thematic analysis [51, 54, 68]. This included data familiarity, initial coding, theme identification, theme revision, and theme definition [51–54, 68]. Memos, transcription, active reading, and re-coding assisted in data familiarity [51–54, 66–68]. NVivo (version 1.0 [2020], QSR International, 2022) was used for initial coding, with the study team collectively coding several initial transcripts to ensure consistency [51–54, 68]. Theme identification occurred often concurrently with coding, with some being identified in memos, and a themebook revised (using both the external heterogeneity and internal homogeneity concept of Patton and level one and level two approach of Braun and Clarke) until no substantive changes were found – at this point final themes were adopted, and then described [51, 69].

## Results

**Fig. 2:**
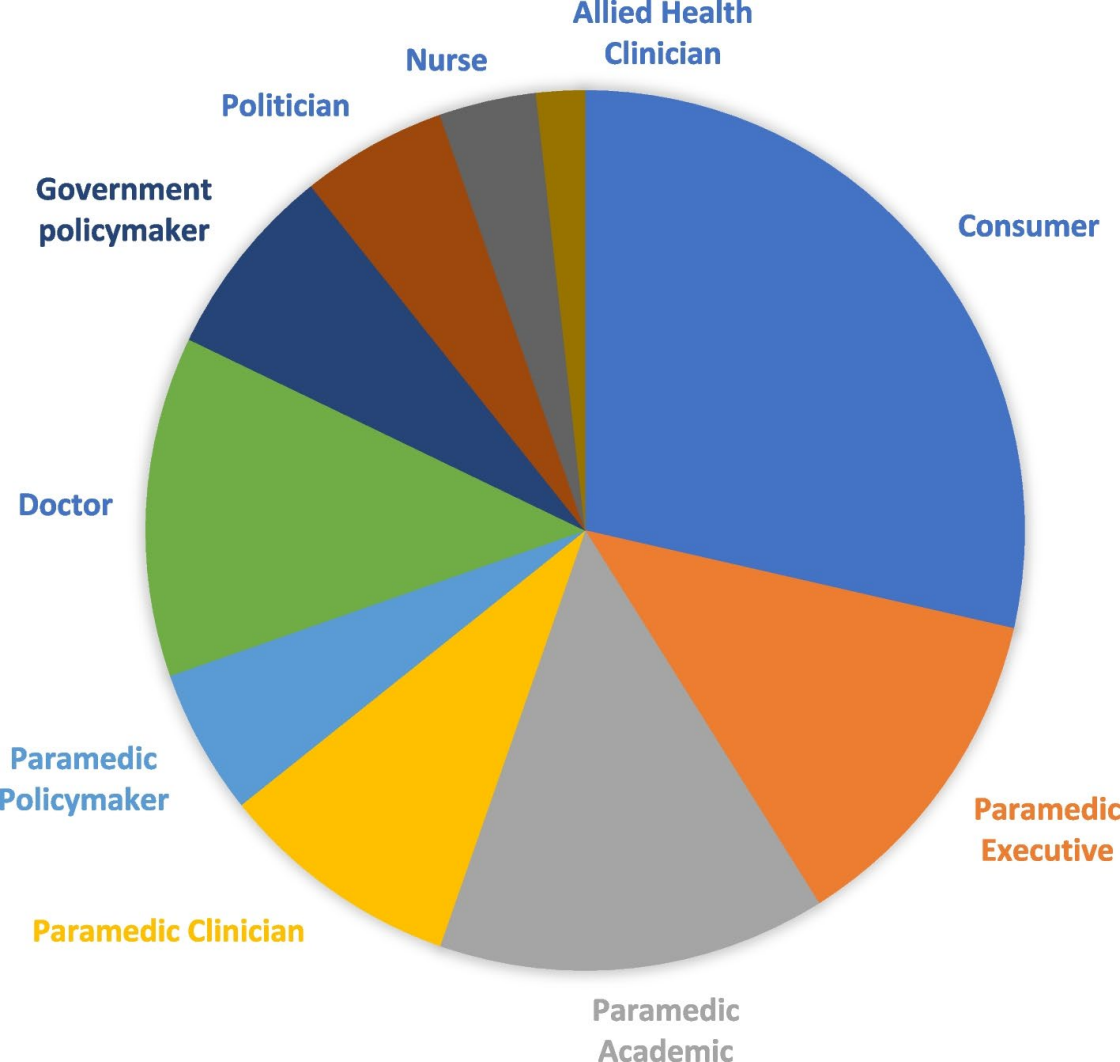
Proportion of participants by background

There were 56 participants, divided into approximate thirds: 29% consumers (*n* = 16), 41% paramedics (*n* = 23), and 30% other health professionals and policy leaders (*n* = 17). The characteristics of these participants are summarised in Table 1, while the proportion of different backgrounds is illustrated in Fig. 2.

**Table 1:** Participants’ characteristics

Participant	Background	Role
1	Paramedicine	Clinician– Paramedic Practitioner
2	Paramedicine	Clinician– Critical Care Paramedic
3	Government Policy	*Withheld*^a^
4	Paramedicine	Academic
5	Government Policy	*Withheld*^a^
6	Paramedicine	Academic
7	Paramedicine	Clinician– Paramedic Practitioner
8	Consumer	
9	Paramedicine	Clinician, Academic
10	Consumer	
12	Consumer	
13	Government Policy	*Withheld*^a^
14	Paramedicine	Academic
16	Consumer	
17	Consumer	
18	Consumer	
19	Emergency Medicine	Clinician, Policymaker
20	Paramedicine	Academic
21	Paramedicine	Academic
25	Emergency Medicine	Clinician
26	Paramedicine	Executive
30	Government Policy	*Withheld*^a^
33	Consumer	
39	Member of Parliament	
41	General Practice	Clinician, Policymaker
43	Consumer	
45	Member of Parliament	
48	Paramedicine	Clinician– Paramedic Practitioner, Academic
49	Paramedicine	Executive
51	Paramedicine	Clinician– Paramedic Practitioner, Academic
52	Paramedicine	Clinician– Critical Care Paramedic, Paramedic Practitioner, Policymaker
53	Consumer	
54	Consumer	
55	Emergency Medicine	Clinician, Policymaker
57	Consumer	
62	Member of Parliament	
63	Government Policy	*Withheld*^a^
64	Emergency Medicine	Clinician, Policymaker
65	Paramedicine	Executive
66	Consumer	
67	Paramedicine	Executive
68	Paramedicine	Academic
70	Consumer	
72	Paramedicine	Academic
74	Consumer	
75	Emergency Medicine	Clinician, Policymaker
78	Consumer	
80	Paramedicine	Executive
81	Paramedicine	Executive
85	Paramedicine	Policymaker
87	Nursing	Clinician– Emergency
88	General Practice	Clinician, Policymaker
91	Government Policy	*Withheld*^a^
94	Consumer	
95	Paramedicine	Academic
99	General Practice	Clinician

The Role column uses generic titles (such as Paramedic Practitioner) for de-identification and consistency, rather than the specific titles each individual may hold

Participants were randomly assigned a number between 1 and 99 for de-identification purposes; these are not sequential

^a^Some participants in this study hold unique senior roles– such as being the only person nationally to have that position– that would make them easily identifiable by explicit description of their role; further information has been withheld for these individuals to maintain anonymity

Four themes were identified. ‘Consumer-centred care, according to consumers*’* reports what consumers desire from their healthcare system and their views on if Paramedic Practitioners meet this. ‘Never heard of it, not sure about it’ captures the perspectives of healthcare disciplines on Paramedic Practitioners. ‘Getting everybody on board’ discusses the barriers and enablers to models identified by participants. Finally, ‘*Are we there yet*?’ compiles participants’ perspectives on how success should be defined and measured for these models. To illustrates themes, a concise set of quotes have been reproduced below. For an expanded compilation of 18,000 words of quotes organised by theme, see Appendix I.

### Consumer-centred care, according to consumers

Consumers described calling ambulance services for non-emergency conditions as they weren’t sure if their complaint is an emergency or not, and that what they want is someone to promptly assess them, reassure them, and solve their problem. Consumers were highly positive about being assessed by Paramedic Practitioners for non-emergency conditions, and saw them as meeting their needs better than alternative options (stated by participants to be either a delayed and brief GP appointment with likely gap fees, or Registered Paramedic transportation to an ED).

It’d actually be perfect… I think it would be a brilliant option. *– Consumer #12*

Yes… The answer is yes. *– Consumer #54*

A short answer would be yes. *– Consumer #94*

Consumers considered ambulance services convenient and reliable in an otherwise confusing and often inaccessible healthcare system, a perspective mirrored by many participants from within paramedicine.

I don’t have a regular GP. No one does. I see someone new for five minutes, they ask five questions, give me antibiotics and send me home. Great if a paramedic can do that in my house, don’t see why they can’t.– *Consumer #10*

I don’t care if I’m transported or not. I’d rather not be. I don’t care if I see a doctor or not. I’m happy with a paramedic.– *Consumer #16*

We can design systems based on arbitrary parameters imposed by statutory ambulance services. Or we can design systems based on community needs. *– Paramedic Executive #26*

### Never heard of it, not sure about it

Outside of paramedicine, Paramedic Practitioners were largely unknown, and several national leaders openly stated that they simply weren’t considered at the highest policy levels.

I have to confess I’m not familiar with the models that are being proposed…

paramedical primary care was not raised and not discussed at any point. *– Government Policymaker #30*

We are 12 years in, and I still speak regularly to other members of my government department, saying ‘We do point-of-care blood testing’; they say, ‘Oh my god I had no idea’.– *Paramedic Executive #1*

As a GP I’m hungry to know this stuff. I actually think you guys should go to the College of GPs and do some education for GPs. *– General Practice Clinician #99*

If you asked most health practitioners five years ago what a paramedic does, they just go, ‘Oh, go to road traffic accidents and cardiac arrests’, and still today that’s all we’re seen as doing by some people. And our work is, extremely, much more complex than that. *– Paramedic Clinician-Researcher #51*

When Paramedic Practitioner models were outlined, a small group of senior policymakers disagreed with their use.

It’s almost a bit outrageous to suggest that they’re going to be primary care paramedics. What the hell can they possibly do? *– Medicine Policymaker #41*

Paramedics shouldn't be stepping into a space that is delivered by primary care… if paramedics keep on providing wound care, antibiotics– if paramedics start giving them contraceptive pills– then we run the risk that paramedics actually then fill in a quasi-primary care role, and I actually don't think that paramedics have the skills to do all that. *– Government Policymaker #3*

It is important to note that these two policymakers had earlier stated that they had no prior knowledge of Paramedic Practitioners, their scope, or the literature examining them. These responses therefore convey their immediate reaction to the concept, rather than a detailed consideration of the evidence. Beyond this small cohort of policymakers, Paramedic Practitioners were viewed positively.

I’m a believer in them.– *Member of Parliament #45.*

I personally think the paramedics have a lot to offer here. *– Emergency Medicine Policymaker #64*

I thought the paramedic specialist idea was really great. *– General Practice Clinician #99*

I think we’d be silly not to take advantage of paramedics and their experience in pre-hospital. *– Government Policymaker #13*

Being a GP is a very hard job, but a lot of the patient presentations in isolation are not that complex. If you’re just doing a repeat script for someone or telling them they have a virus and they can go home and run a medical certificate. You do not need to be a GP to do that. I did that when I was a medical student. *– Emergency Medicine Clinician #25*

This is obviously a fraught area to address. There probably needs to be a conversation about what represents an acceptable quality of care… I wonder if we need to accept that a well-governed system of other kinds of health care professionals delivering something in that primary care space can be better than a system that says, “No, it can only be GPs, and yet we don’t have enough GPs, and we also accept that a portion of the GP workforce is not delivering very good care.”– *Paramedic Policymaker #65*

[Paramedic Practitioners should have] prescribing rights for basic things as well… if someone’s got just like a mild cellulitis or community acquired pneumonia. *– Emergency Medicine Clinician #25*

I like that model, because if I look at my practice, you know, I’m literally booked out ‘til end of June… I love them. You know, in GP land, they’re actually a source of learning. I think there’s two-way learning that goes on when you have GP, paramedic, ambulance services meeting over the top of a patient. *– General Practice Clinician #99*

I think the professions have got to show more professionalism and say, well, if doctors are burned out and doctors are up to their neck with workload… then what is it that they shouldn’t do? What takes pressures away from them? *– Member of Parliament #62*

All healthcare professionals agreed that the complexity of non-emergency consumers was under-recognised, particularly among the broader paramedic cohort who historically have only had the sole step of career progression to critical care.

100% paramedics need to be more aware that these are complex patients. *– Paramedic Clinician-Researcher #51*

There is a narrative out there that the things that get done in community medicine must be simpler because they don’t require a hospital in order to achieve good outcomes, and I can’t say that anything could be further from the truth. *– General Practice Policymaker #41*

I’m a Physician Assistant also, which is a Master’s degree in primary health care. I’m also a Critical Care Paramedic. My primary health care skillset took three years, and it’s just incredibly, much more complicated and difficult than the critical care. On reflection the critical care was really easy. So the education is really critical, and I would say at least to be at a Master’s level. *– Paramedic Clinician #51*

### Getting everybody on board

The third theme captured the wide variety of opinions on what makes Paramedic Practitioner programs succeed or fail. Frequently mentioned was development of a reliable education program.

I think it’s a really necessary part of the puzzle. But the barriers here to it, currently, I’d say is a trusted educational program.*– Emergency Medicine Policymaker #19*

In many universities it’s not even a viable course, because you’re competing against large ambulance services that do the courses in-house. *– Paramedic Academic #68*

Adoption of a rotational model, where paramedics work partially in ambulance services, partially in EDs, and partially in GP clinics, was widely praised for breaking down professional silos and maintaining clinician capabilities.

I love the rotational model. I love the idea of a paramedic being attached to my GP clinic. *– General Practice Clinician #99*

I think there’s an absolute value for paramedics doing 50% in urgent care, 50% on the road because you keep those skills, and you keep the primary care. *– Paramedic Manager #67*

Having Paramedic Practitioner scope tailored to local community needs to augment rather than to cannabalise existing services was viewed as essential.

The first six months is just spent finding what's needed and then targeting our services to match what's needed, and that's why all of our Paramedic Practitioner roles are different within the State. *– Paramedic Clinician #7*

Every community will be absolutely different. *– Government Policymaker #5*

This isn’t one cohort. This is lots and lots and lots and lots of little different cohorts. *– Paramedic Clinician-Researcher #51*

Co-design, co-design, co-design.– *Consumer #70.*

Similarly, relationships with local primary care providers were considered key to success.

What you’re wanting to do is upskill the emergency department about what the paramedics’ skills are, and the GPs as well. *– General Practice Policymaker #88*

There needs to be buy in from the other people who are doing that work. *– Government Policymaker #91*

On-demand medical specialist consultation was viewed as beneficial for safety and sharing accountability for complex cases, particularly in areas where Paramedic Practitioners were being newly introduced and not yet established.

Anything with telehealth. As long as you have access to a supervisor, so that they don't work outside their scope of practice, anything like that is very safe, and has been very well proven. *– Emergency Medicine Clinician #25*

Development of formal referral pathways were recommended, with examples given by participants including fields as varies as pharmacy, psychology, and even dietetics.

You can refer them to the physiotherapist, occupational therapist, or whatever it might be. *– Government Policymaker #3*

Rural areas were noted as a unique environment that required broader scope, more access to specialist consultation, and separate outcome measures.

Paramedics in rural areas and underutilised… some remote stations might do two to three jobs are week at best. We could get a lot from that paramedic doing preventative primary health care within that community. *– Paramedic Clinician #2*

Finally, paramedics discussed the ongoing perception from within the profession of Paramedic Practitioners as being disregarded in favour of ongoing focus on critical care.

A system where the paramedics are valued and highly trained. This should be our best people, and this should be seen as this Paramedic Practitioner pathway is at a similar footing to an intensive care paramedic .*– Paramedic Clinician #2*

A total of 10 enablers to the success of Paramedic Practitioner models were raised:

1. Appropriate Paramedic Practitioner training, including a trusted national qualification at Master’s level provided by universities and national regulation;
2. Use of a rotational model between ambulance services, GPs, and EDs;
3. Local variations in scope of practice according to community needs;
4. Building relationships and augmenting (rather than cannibalising) local primary care providers;
5. Access to on demand specialist consultation, including GP and ED physicians, occupational therapy, social work, psychology, and pharmacy;
6. Formalised referral pathways and referrals beyond GPs to Allied Health;
7. Unique models, funding, and performance indicators for rural and remote services that recognise a lower level of efficiency is ethically acceptable for redressing inequalities in health outcomes;
8. Including Paramedic Practitioners as part of multidisciplinary teams;
9. Ensuring smooth transitions of care with complete and point-of-service paperwork handover; and,
10. Good governance and long-term investment.

A series of barriers were also identified. Within paramedicine, Paramedic Practitioners were thought to often be inappropriately dispatched by ambulance services to emergency jobs in order to ‘stop the clock’ and assist in meeting government key performance indicators related to response time– a measure stated by several participants as not being correlated to better consumer outcomes.

They get used as clock stoppers… their work is so often subsumed by operational demand. *– Paramedic Clinician #9*

[*Sarcastic*] Seriously? Like, oh, we got there so quickly, well done. Tick. *– Paramedic Academic #48*

Several participants from policy backgrounds discussed a lack of national practice standards for Paramedic Practitioners leading to concerning unreliability in education and capabilities, with the potential to impact consumer safety.

We need practice standards to be set, and the best place for that to occur would be from the Paramedicine Board. *– Government Policymaker #5*

It's very nice to have a defined scope of practice for an emergency department. *– General Practice Policymaker #41*

The inconsistent terminology used for Paramedic Practitioners was noted as causing confusion for those outside of paramedicine, who were unsure which roles were which.

We have some language which needs to be firmed up. Very, very keen to see that done by Ahpra. *– Government Policymaker #5*

Nomenclature, it’s all a bit confusing across the jurisdictions. *– Government Policymaker #13*

Ongoing professional silos were widely discussed, particularly the ongoing segregation of paramedics from the wider healthcare system.

I mean, look at the UK, they’ve got paramedics working in hospital departments. We don’t do that here. It is that siloed approach. *– Paramedic Academic #68*

I’m not sure that the ambulance services have traditionally engaged very well with others. Whilst they’re trusted by the community, they’re not trusted by pretty much anyone they have any business dealings with. *– Government Policymaker #5*

The most frequently and passionately discussed issue was for increased access to medical records, including both access prior to or during treatment, and input of paramedic records in real time. Those outside of paramedicine were shocked that paramedics do not universally have prospective access to consumer medical records.

I go to my GP a week later and they have no idea that the paramedics came, that I was in hospital. I have to try and remember. I don’t know about the names of things, the medications. I can’t remember. They need to talk to each other, not put it on me. – *Consumer #16*

I can’t believe… I’m both surprised and not surprised by that… really shocks me. *– General Practice Clinician #99*

I want paramedics to see my medical records! What if I’m unconscious? ‘000’ is the most important time for anyone to see my records. That’s ridiculous. *– Consumer #10*

The double talking, the double doing, double double double! What’s his, Henry Ford? Didn’t he invent the assembly line? He’d be having a seizure. He’d think we’re insane. *– Consumer #33*

National datasets– particularly the Productivity Commission’s Report on Government Services (ROGS)– were widely dismissed by policymakers and executives as being based on inconsistent reporting and historic metrics that are improperly measured and no longer relevant, inhibiting informed decision-making.

The Report on Government Services, it calls itself an experience survey. It's actually just a satisfaction survey. It's not validated. It's not psychometrically tested and interestingly, it's produced by the Council of Ambulance Authorities. Funnily enough, the same people that provide the service. So you can't tell me there's no bias there. That’s used as a reportable government service output. How terrible is that? *– Paramedic Clinician-Researcher #51*

ROGS data is terrible. *– Government Policymaker #5*

The ROGS data has really not kept up. – *Paramedic Executive #26*

The funding model of several ambulance services was noted to remain tied to their historic mandate of transportation, having not kept up with advances in the profession, and that this financially disincentivised managers from promoting consumer-centred care-in-place.

[Services are] paid to transport people to hospital. So why would you, as an ambulance service then go, “Hey? Let's like not take people hospital. Let's take them to where they need to be, or advise them appropriately”… It doesn't make sense. *– Paramedic Clinician-Academic #48*

All of the emergency ambulance services will see a reduction in their annual income as a consequence of that. *– Chief Officer #5*

A total of 10 barriers to Paramedic Practitioner models’ success were raised:

1. Managers and policymakers being indifferent or uninformed about these models;
2. Inappropriate dispatch of Paramedic Practitioners to ‘stop the clock’;
3. A lack of national practice standards for Paramedic Practitioners;
4. Inconsistent terminology;
5. Ongoing professional silos limiting consumer-centred care;
6. No paramedic access to medical records, and no or belated subsequent sharing of paramedic records;
7. National datasets that lack rigor, are unreliable, and collect metrics not reflective of contemporary ambulance service issues;
8. Funding models that reward transportation to the ED, and consequently disincentivise care-in-place by Paramedic Practitioners that results in a loss of ambulance service income;
9. Funding disincentives that don’t support Paramedic Practitioners; and,
10. Primary-urgent services that aren’t available out-of-hours to align with continual consumer presentations.

### Are we there yet?

The final theme captured all discussions about how outcomes should be measured. Multiple outcomes were considered necessary. However, there was significant conflict between participants as to which outcomes are appropriate, and many senior policymakers were unsure which outcomes would be meaningful.

I think you will need 30 measures? No, 10? No, I don't know how many. *– Paramedic Policymaker #85*

Economic evaluations… there needs to be a robust framework for economic evaluations of these models of care. *– Paramedic Academic #95*

I would want to know if there’s any adverse outcome, those near misses. – *Emergency Medicine Policymaker #75*

What would be deemed an adverse event from this? I'm not sure. – *Chief Officer #30*

I definitely think that the health economics is a really important and largely missing piece. *– Paramedic Executive #26*

Patient satisfaction– but that would be likely to be high. – *Emergency Medicine Policymaker #55*

We want to bring joy to everyone, right, in their workplace? So is this good for paramedics? *– Consumer #66*

Re-presentations would be a good measure. *– Emergency Medicine Clinician #25*

I see a lot of models with KPIs around diversion and around the money, things can get a bit skewed, because you divert people to nowhere. – *Emergency Medicine Policymaker #64*

Are you doing meaningful work? *– Paramedic Academic #21*

We've delivered an astounding health service, and I might not have given one medication. I might not have done any paramedic intervention, but purely listening to, assessing and providing advice, I've given that person good health care. And that needs capturing. *– Paramedic Clinician-Researcher #51*

The outcomes raised included:

1. Appropriateness of care;
2. Consumer safety and health outcomes;
3. Economic impact;
4. Consumer experience;
5. Practitioner experience; and,
6. Efficiency impact (including re-presentation rates and ED transportation rate).

## Discussion

This study has sought to answer the question: what are the perspectives of interest-holders on reactive Paramedic Practitioner models within Australian ambulance services? Despite the use of precursors to Paramedic Practitioners for over 33 years globally and 18 years in Australia, a correspondingly significant amount of published research including over 150 articles specifically on reactive models within ambulance services and numerous Australian government and industry reports, some of Australia’s most senior national healthcare figures were unaware of the existence of these models, and stated that they have simply not been represented in policy discussions. This presents a major barrier to informed discussion of these models. Previous research on this topic has shown several frameworks to understand policy change; most contain a stage at which a sufficient convergence of interests among interest-holders and decision-makers is achieved [46–48]. This most prominently includes the multiple streams framework of convergence between policy problems, policy solutions, and politics [46, 47]; the triangle of context, content, and process [70]; or those originally pioneered by Weiss [71], among dozens of others [72]. Additionally, due to path dependence (the ‘stickiness’ of institutions to existing practices), policy change is recognised as difficult [73]. Therefore, research is rarely directly translated into practice, but instead slowly influences opinions in policy until a ‘window’ to enact change arises [46–48]. This study shows that high-level policy decisions– such as the Medicare Taskforce– are reportedly largely made without consideration of paramedicine, and consequently a convergence of interests to fully consider these models is not possible. Therefore, the most important finding of this study is that introduction, evaluation, and integration of Paramedic Practitioners requires, first and foremost, awareness among the broader healthcare community.

There is a disconnect between perspectives of consumers and senior healthcare policymakers that was, in some instances, stark. While the appropriate application consumer-centred care remains a source of ongoing study [74–76], this disconnect is nonetheless suggestive of a healthcare system that is not meeting the needs of its funders and users. Consumers reported calling ‘000’ because of uncertainty about whether they were having an emergency or not and the difficulty in accessing the wider healthcare system; they saw Paramedic Practitioners as providing safe, rapid, and convenient care that met their needs. Several senior healthcare policymakers, however, strongly favoured maintaining a segregation of professional roles largely due to safety concerns about any care delivered by non-physicians, a topic most widely researched in the medical-nursing relationship context [77–79]. These participants suggested that consumers should be required to seek primary care from the general practice cohort, despite the widely-reported increasing gap fee expense, prolonged appointment wait times, and short appointment length that consumers– particularly marginalised groups– have reported as affecting their access to and quality of care [80–86].

A surprising finding of this study that we were unable to find previous research investigating was that there was no difference in opinion on Paramedic Practitioners based on clinical background—paramedics, nurses, and doctors all shared similar views. Differences instead arose based on perspective; those from a high-level national or state policy perspective had a perceived low risk tolerance and preferred clear delineation of roles, while frontline clinical staff strongly supported a'mixing' of different roles to address community presentations and felt the associated risk was appropriate. From a realist lens, one interpretation of this may be that the role of an individual shapes their opinion more than their clinical background: frontline staff may be motivated to pursue what they see as practical solutions to the immediate problems they directly encounter on a daily basis, while senior policy staff instead take a relatively sterile view that seeks to balance broader systemic considerations. However, a notable exception to this were the parliamentarian participants in our study, all of whom expressed views more closely aligned with the frontline staff– but who additionally stated that enabling change from their legislative role was limited by the political cycle.

### Translation of research into practice

For ambulance services using Paramedic Practitioner models, consideration should be given to the 10 enablers and 10 barriers identified to allow shared learning and maximise the benefit of these models. This study also specifically outlines– from a broad base of interest-holders– what outcomes are of interest to them when forming their policy position. Using a standardised set of outcomes known to be of interest is encouraged [87], and this study provides a list of six outcomes specifically sought by policymakers on this topic. Researchers may wish to consider this list when designing future studies.

## Limitations

Several limitations should be considered by users of this study. Firstly, the aim and methodology adopted here do not assess objective outcomes, and consequently do not indicate that Paramedic Practitioners are beneficial. Outcomes from these models are thoroughly reported in over 150 academic papers elsewhere [13, 28]. This study instead seeks to report the perspectives of key interest-holders in the Australian healthcare system. Secondly, the study aimed to gain breadth of viewpoints rather than depth, and the perspectives of interest-holders may not always be representative of the entire community that they represent, such as survey responder bias for consumers or clinician participants. Thirdly, all comments by participants are taken as stated. This study did not seek to test any statements, and their accuracy is not investigated. Similarly, the method assumes that participants are truthful and candid about their beliefs. This research did not investigate all possible uses of Paramedic Practitioners (such as their role in GP clinics, EDs, or urgent care centres) or consider proactive models; viewpoints provided are exclusively on these clinicians operating reactively within ambulance services. Finally, there were no consumers within the study team, and while several professions were represented (paramedicine, nursing, and medicine), there is the possibility that this impacted interpretation. Mechanisms to mitigate this are discussed above under Methods.

## Conclusion

This study reports interest-holders perspectives on Paramedic Practitioners within Australian ambulance services. There was widespread support for Paramedic Practitioners from consumers, paramedics and other health professionals, with a small but avidly dissenting contingent of national policymakers. Four themes were identified, with 10 enablers, 10 barriers, and 6 outcomes of interest– these results of this study can be used by policymakers, managers, and researchers when evaluating Paramedic Practitioners within ambulance services.

## Supplementary Information

Supplementary Material 1.

## Data Availability

The Supplementary Materials contains three appendices. Appendix I contains a deidentified selection of quotes to illustrate themes and sub-themes. Full transcripts are not intended to be stored in a public repository as, despite undergoing deidentification, the highly unique positions of some interviewees means that reidentification remains a concern. Appendix II contains the open consumer recruitment advertisement used. Appendix III contains the initial iteration of the semi-structured focus group protocol.

## Abbreviations

Ahpra: Australian health practitioner regulation agency
CEO: Chief executive officer
ED: Emergency department
GP: General practitioner
LNR: Low or negligible risk
ROGS: Report on government services
UK: United Kingdom
