# Supplementary material for: Paramedic Practitioners within ambulance services: views of Australian policymakers, health professionals, and consumers
Source: BMC Health Serv Res. 2025 Apr 10;25:533. doi: 10.1186/s12913-025-12614-y (PMC11987361; doi:10.1186/s12913-025-12614-y)
Supplement: Supplementary file 1 — Supplementary Material 1. [file 12913_2025_12614_MOESM1_ESM.pdf]

Appendix I: Additional participant quotes organised by theme and sub-theme

Quote attribution: - *Participant number, Background (Specialty), Role (Jurisdiction)*

Patient-centred care, according to patients **2**

Never heard of it, not sure about it **3**

Lack of awareness 3

Disagreement at high policy levels 3

Broad support 4

Acknowledging complexity 7

Getting everybody on board **8**

Appropriate paramedic training 8

Use of a rotational model 10

No one size fits all: there must be local variations 10

Don't go it alone: building relationships with local primary care providers and patients 12

Access to on demand specialist consultation 13

Referral pathways 15

Unique models, funding, and performance indicators for rural and remote services 16

Viewpoints on specialist paramedics as part of multidisciplinary teams 18

Ensuring smooth transitions of care 18

Good governance and long-term investment 18

Managers and policymakers aren't interested 19

Inappropriate dispatch of specialist paramedics 19

A lack of national practice standards for specialist paramedics 20

Inconsistent terminology causing confusion 21

Professional silos 22

No access to medical records, no sharing of medical records 23

Data that is unreliable or not shared inhibiting informed decision-making 27

Funding models that act as disincentives to non-transport 28

Funding disincentives that don't support specialist paramedics 28

Primary-urgent services that are available out-of-hours 29

Are we there yet? **30**

Multiple outcomes are necessary 30

Appropriateness of care 30

Cost 31

Safety 32

Patient experience 32

Practitioner satisfaction 33

Re-presentation rates 34

ED transportation rate by specialist paramedics 34

Miscellaneous **35**

Patient-centred care, according to patients

It'd actually be perfect... I think it would be a brilliant option. – *12, Patient*

Yes... The answer is yes. – *54, Patient*

A short answer would be yes. – *94, Patient*

I am ok with not being transported to a hospital or the ED after calling 000. – *17, Patient*

I don't have a regular GP. No one does. I see someone new for five minutes, they ask five questions, give me antibiotics and send me home. Great if a paramedic can do that in my house, don't see why they can't. – *10, Patient*

I'd probably be comfortable with that. The expectation, from my point of view, would be at least to do some triaging, basic vital signs check, then really go from there to determine if or not there is a need for further investigation through emergency. – *94, Patient*

I don't care if I'm transported or not. I'd rather not be. I don't care if I see a doctor or not. I'm happy with a paramedic. I have no idea if it's an emergency and I need someone to tell me what to do. – *16, Patient*

The situation where I've been in is usually where I'm feeling like I'm going to vomit. So if a paramedic got me over that, I'd be fine. – *54, Patient*

I would probably be upset... I just need that reassurance [of going to hospital]. – *70, Patient*

For health consumers, when we poll health consumers, which we do regularly, and ask them what the number one thing they want is they want affordable, fair, timely access to primary health care. That's what Australians want more than anything... They want access to that person that they can afford. They can get it when they need it, and that it's fair. – *66, Patient Representative*

People have completely forgotten what 000 is for. 000 is not for primary care, it's not for urgent care, it's for emergency care. The problem is people are now using 000, or misusing 000 in a way it was never designed to do. – *18, Patient*

Having somebody who can come out to you and just have that physical connection... a health trained person who can come to your home and then have that chat. – *70, Patient*

What consumers want is one interface. – *66, Patient Representative*

Have we asked consumers what they want? And if so, have we asked them how they want it? – *66, Patient Representative*

I would be okay with that as a patient. It should be a bit more up to the paramedics to make that sort of decision rather than just like their policies and procedures that they have to follow and things like that. Because they're, you know, well trained professionals, if they believe that this can be managed by a GP visit then they should be able to make that decision if the patient likes it or not. And as a patient, I would be okay with that. – *57, Patient*

I didn't even know that existed. I just, you know, your normal paramedic, and then flight care paramedic. That's all I know. So, I didn't know that there was such thing. – *74, Patient*

More and more people expect an interface to be very clever, and that there may be a lot of players sitting at the back that are doing their thing. – *66, Patient Representative*

Never heard of it, not sure about it

Lack of awareness

I have to confess I'm not familiar with the models that are being proposed… The Strengthening Medicare Taskforce is the direction in which government is going… paramedical primary care was not raised and not discussed at any point during that meeting. – *30, Chief Officer (National)*

As a GP I'm hungry to know this stuff. I actually think you guys should go to the College of GPs and do some education for GPs on, in your patient cohort, this many are presenting to hospital with Category 5 presentations. – *99, Medical (GP), Clinician*

Breaks down those barriers very early to open up conversations and understand each of them and we're human beings. When we understand people, we tend to work much better with them, rather than fight them away. – *51, Paramedicine, Clinician-Researcher*

We are 12 years in, and I still speak regularly to other members of my government department, saying 'We do point-of-care blood testing'; they say, 'Oh my god I had no idea'. – *1, Paramedicine, Manager*

If you asked most health practitioners five years ago what a paramedic does they just go, "Oh, go to road traffic accidents and cardiac arrests", and still today that's all we're seen as doing by some people. And our work is, extremely, much more complex than that. – *51, Paramedicine, Clinician-Researcher*

Disagreement at high policy levels

It's almost a bit outrageous to suggest that they're going to be primary care paramedics. What the hell can they possibly do? – *41, Medicine (GP), Policy (State)*

You're just setting these people up to fail. And are they going to be the next health system scapegoat? – *41, Medicine (GP), Policy (State)*

Paramedics shouldn't be stepping into a space that is delivered by primary care… if paramedics keep on providing wound care, antibiotics – if paramedics start giving them contraceptive pills – then we run the risk that paramedics actually then fill in a quasi-primary care role, and I actually don't think that paramedics have the skills to do all that. – *3, Chief Officer (State)*

I also am concerned a little bit about, without being derogatory about patients at all, sometimes these kind of services can then reward patients who are addressing their needs on, you know, single episode of care, rather than preventive and rather integrated medicine. – *41, Medicine (GP), Policy (State)*

We would say that for a GP really to be starting to be, you know, expert in their field, they would have to have been fellowed for 10 years. – *41, Medicine (GP), Policy (State)*

It concerns me that certain medical problems can be made to seem simple when often they're not… even just prescribing - there's no drug that isn't a poison. So when I hear people sort of talking about simple models of you know, UTI prescribing, that's got risk and problems written all over it from my point of view. And it horrifies me that other people can't understand the complexity of it. – *41, Medicine (GP), Policy (State)*

You're supposed to deliver to them some kind of solution - it's actually not going to be possible. – *41, Medicine (GP), Policy (State)*

People that are going to interact with the ambulance system in this way are more likely than not to have

the most complex needs, to have their needs intersecting with problems from a psychosocial point of view, potentially drug and alcohol point of view, certainly mental health issues, and they are the ones that experience the most fragmented care. And this model is going to suggest that fragmenting care is going to solve a problem. – *41, Medicine (GP), Policy (State)*

I'd be a bit mindful there, because there there's obviously a huge issue of scope creep in that space. – *55, Medicine (Emergency) Policy & Clinician*

It's solving a problem from the government's point of view, and from, you know, ambulance use point of view. But I don't know if it's actually going to solve a problem from the patient's point of view. – *41, Medicine (GP), Policy (State)*

We're trying to solve a really dire systems problem with solutions that don't address what's going on underneath the surface. – *41, Medicine (GP), Policy (State)*

I have no doubt that paramedics could pick up and learn whatever we want them to learn. It's just a matter of where it's best placed. – *64, Medicine (ED), Policy (National)*

Patients will find it convenient; they will find any service that can be provided to them inside their house without a cost will be one that it's inevitable will be taken up. It's just a question of whether it's actually in the best interest for the patient, and whether there's the ongoing downstream effects. – *55, Medicine (Emergency) Policy & Clinician*

Are we simply plugging the hole in the dam with another model, rather than necessarily addressing the fundamental issue of access to primary health care for our community more broadly? – *30, Chief Officer (National)*

You have to be mindful of any model that actually has downstream consequences, that is actually not helpful. One of the things within the urgent care service model, for example, is that there will be a cohort that uses that as the default primary care service, and don't go back. Having that ongoing continuity of care within their primary provider so, you know, there's a risk there that if the paramedic is providing their primary care instead of the patient going back and following up and getting things checked up, they don't get the longevity. That can have more downstream effects. – *55, Medicine (Emergency) Policy & Clinician*

Broad support

Is the idea of paramedics doing this kind of stuff, this non-resuscitation stuff where they're moving beyond their traditional role, is that concerning for you? – *Facilitator*
No. – *25, Medicine (ED), Clinician*
What's your initial reaction? – *Facilitator*
I think it can be done. – *25, Medicine (ED), Clinician*

Being a GP is a very hard job, but a lot of the patient presentations in isolation are not that complex. If you're just doing a repeat script for someone or telling them they have a virus and they can go home and run a medical certificate. You do not need to be a GP to do that. I did that when I was a medical student. – *25, Medicine (ED), Clinician*

This is obviously a fraught area to address. There probably needs to be a conversation about what represents an acceptable quality of care. I don't doubt that GPs do amazing work, and a really experienced, highly educated, passionate GP can offer every single patient a wonderful thing. But, I think we also accept that not every patient is ever going to have access to the highest performing members of the GP population. We accept currently that there's a huge cohort of general practitioners who are not,

by definition, the highest performing end of the cohort. I suspect people in the emergency care world are frequently encounter pretty suboptimal care delivered by a part of the GP workforce. And I wonder if we need to accept that a well-governed system of other kinds of health care professionals delivering something in that primary care space can be better than a system that says, “No, it can only be GPs, and yet we don’t have enough GPs, and we also accept that a portion of the GP workforce is not delivering very good care”. Can we do better? Albeit not the ideal situation. But can we do better by having a different part of the health care workforce delivering something in that primary care space. – *65, Paramedicine, Manager*

I think the professions have got to show more professionalism and say, well, if doctors are burned out and doctors are up to their neck with workload… then what is it that they shouldn’t do? What takes pressures away from them? – *62, Parliamentarian*

I’m a really strong advocate for paramedics that have not just emergency skill set, but a primary health care skill set and the capacity to deliver care such as, suturing, and you know some antibiotics. That requires a skill set and I think that's what will change, is that paramedics will increasingly be involved with primary health care, because that's the workload. – *52, Paramedicine, Policy (National)*

I think that is needed to address the low acuity demand. I’d build upon that if I was running an ambulance service. More [community paramedics], free up our ambulance crews. – *45, Parliamentarian*

I personally think the paramedics have a lot to offer here. Okay, you know, I saw someone with conjunctivitis, just brought in by to hospital on Monday night, and I’m sort of like, oh, this could have just been dealt with by the paramedics. That’s not currently allowed in the system. – *64, Medicine (ED), Policy (National)*

Someone who just needs a catheter change, does not need an ambulance crew, an emergency ambulance crew, taking that dementia patient who is going to be frightened anyway, in the middle of the night, to go and get a catheter changed. – *45, Parliamentarian*

If we look at various international models, this is where the primary healthcare paramedic can possibly fit in and be able to treat, do home based care, be able to look at different referral pathways for their patients, get them started and add a course of antibiotics and then do a referral, make an appointment up with the GP. – *72, Paramedicine, Academic*

This is the missing middle. – *99, Medical (GP), Clinician*

I don't think adding more people to the primary health care service is the answer. I think it's about having a look at what those gaps are, and some of those gaps are actually best suited with ambulance. – *81, Paramedicine, Manager*

I think there’d be some people who would be concerned by it. – *25, Medicine (ED), Clinician*

There’s a bit of a like a lost bracket from like 55 to 65, where people aren’t eligible for an ACAT assessment and aged care, right? But if you’ve been doing heroin and meth your whole life, and you smoke 40 cigarettes a day, you are 70 years old, and you cannot look after yourself, and you may not be able to get yourself to a GP. So having someone who can kind of bridge that gap. – *25, Medicine (ED), Clinician*

Where someone needs an urgent response that can't be delivered by a GP, but doesn't require transport, there is some role there. – *3, Chief Officer (State)*

We have such fantastic skills of always working on the away ground, of always being out of our comfort

zone; I think we would be mad not to apply that. – *1, Paramedicine, Manager*

[Referring to paramedics leaving people at home] That makes me less afraid than having all these people in my waiting room. – *25, Medicine (ED), Clinician*

The co-responder mental health as well, diverting people away. Good evidence there… Those numbers spoke for themselves. – *45, Parliamentarian*

Have a chat with SMOs, Nurse Practitioners in Fast Track. They absolutely love the primary care type paramedic, hands down. – *51, Paramedicine, Clinician-Researcher*

I'm open to all ideas and options. No one's closed to ideas. – *30, Chief Officer (National)*

I have a lot of experience working with people with extended scope of practice, who kind of just work parallel to me. It would be very good to just have a single clinician like, even if they have, I don't know if there's an avenue for like prescribing rights for basic things as well. Because like nurse practitioners, I don't know if the physio's do, but nurse practitioners have a prescribing number, and if someone's got just like a mild cellulitis or they've got, you know, community acquired pneumonia, and they've called out, you know, wanting to go to hospital, but like really, they don't need to go to hospital because their obs are like plumb normal. Or you know you just need to treat their cellulitis, and they can go and see their GP tomorrow. I think there'd be a lot of avenue for actually giving, like having some packs and starting someone on a course overnight. – *25, Medicine (ED), Clinician*

Any service like this research about paramedics who can make that clinical decision before they end up being driven in an ambulance is amazing. – *99, Medical (GP), Clinician*

Typically ambulance has always been a can do type job. Until we get told we can't do it, we'll do it. And unfortunately, well, fortunately, the bureaucrats haven't caught up with us doing that yet, but one day they will. – *7, Paramedicine, Clinician*

I think paramedics are highly skilled, and whenever I've had to call someone to help me out they have been an absolute god send. I love them. You know, in GP land, they're actually a source of learning. I think there's two-way learning that goes on when you have GP, paramedic ambulance services meeting over the top of a patient. – *99, Medical (GP), Clinician*

We can design systems based on arbitrary parameters imposed by statutory ambulance services. Or we can design systems based on community needs. – *26, Paramedicine, Executive*

I think we can change this by providing healthcare in the home. – *19, Medicine (ED), Policymaker (State)*

We had one particular lady who would present 2 to 3 times a month with chest pain, shortness of breath, hypertension. We took her a set of scales, a blood pressure machine, blood glucose machine. We took that round to her, we trained her to use it. From 2016 when we started her on the system until I left at the end of the year she had had one presentation to hospital. So from 4 a month to one in six years. – *7, Paramedicine, Clinician*

I probably think a more specialised group is a better idea. – *2, Paramedicine (Intensive Care), Clinician*

I think we'd be silly not to take advantage of paramedics and their experience in pre-hospital. I think that there is absolutely scope for it, the ECP role. – *13, Government, Policymaker (State)*

I thought the paramedic specialist idea was really great. Because you do need that medical eye to sort

out, yeah, this is a newborn who hasn't fed for 24 hours, and hasn't had any wet nappies. Yeah, that child does need to go to hospital, and are they safe to go in a private car. – *99, Medical (GP), Clinician*

I like that model, because if I look at my practice, you know, I'm literally booked out 'til end of June. – *99, Medical (GP), Clinician*

I'm a believer in them. – *45, Parliamentarian*

Acknowledging complexity

The person, the human being that has asked for health care is very different, and their needs as an individual are extremely specific to them. Their own vulnerabilities are very particular. – *51, Paramedicine, Clinician-Researcher*

I see my role as not necessarily fixing it and then tomorrow them having the same problem, and then me going to them again tomorrow. It's more going, "Okay if we fix it today. But let's empower you to manage this better in the future." – *51, Paramedicine, Clinician-Researcher*

100% paramedics need to be more aware that these are complex patients. – *51, Paramedicine, Clinician-Researcher*

It's a misnomer about ED being simple as well like, we just don't do the immediate stuff – *64, Medicine (ED), Policy (National)*

So I could manage cellulitis at home more than easily myself. But there are people in our community for whom cellulitis should be managed in hospital. – *5, Chief Officer (State)*

It's more, the chronic care plans, and like the knowing when to call people back, and went to some people to the ED, which is where they'd need the actual extra support, I think. – *25, Medicine (ED), Clinician*

The highest unexpected morbidity in an ED patient group is in Cat 4. – *64, Medicine (ED), Policy (National)*

There is a narrative out there that the things that get done in community medicine must be simpler because they don't require a hospital in order to achieve good outcomes, and I can't say that anything could be further from the truth. – *41, Medicine (GP), Policy (State)*

If you have very limited social support, and particularly if you have low socioeconomic status and limited heating, cooling, living conditions, your attendance at hospital actually might be completely appropriate for something that could be managed in community if you had higher level of health literacy, financial means, and or capacity. – *5, Chief Officer (State)*

Getting everybody on board

Appropriate paramedic training

I'm a physician assistant also, which is a Master's degree in primary health care. I'm also critical care paramedic. My primary health care skillset took three years, and it's just incredibly, much more complicated and difficult than the critical care. On reflection the critical care was really easy. So the education is really critical, and I would say at least to be at a Master's level. – *52, Paramedicine, Policy (National)*

I'll imagine, at some point, we're going to end up with that Master's level. That's really only a matter of time, I would suggest. – *6, Paramedicine, Academic*

The one workshop I got the most out was a GP who from head to toes she did an analysis of what every person should have done on them. So, it was about everything you could think of, and then differential diagnosis in the way of does this person live with this all the time, and this is their base. – *81, Paramedicine, Manager*

In many universities it's not even a viable course, because you're competing against large ambulance services that do the courses in-house. – *68, Paramedicine, Academic*

It is a specialty. In the way we're describing it, it is beyond your entry to practice paramedic level which needs to have greater capacity in this space. – *5, Chief Officer (State)*

Emergency resuscitative type response – this is a very, very different approach to delivering medicine, and I think that foundation needs to be acknowledged, but then the gaps need to be identified, and people are trained appropriately for the more primary care type jobs. – *51, Paramedicine, Clinician-Researcher*

It depends on the ambulance services, what model they want to go with respect to postgraduate education versus in-house training. – *6, Paramedicine, Academic*

[Regarding in-house education] The biggest challenge is watching the course being created by the ambulance service was the lack of input, from people experienced in the role. So I feel the voices of the people that were actually working in that role weren't listened to with design of education program. So there's a lot we're trying to say. "Hang on! There's some big gaps here that we don't understand, and that's what we'd like to learn about" But that wasn't listened to. – *51, Paramedicine, Clinician-Researcher*

The right education also helps to make it successful, so they've got the competence and confidence to do the role. – *48, Paramedicine, Clinician-Academic*

I don't think it's all on the university sector to do this paramedic uplift, you know. It's a partnership between service providers at all different levels, private and public, and the university sector to understand what the best educational framework is to meet the changing needs, and that's going to require regular review and change over time. Clearly, there's an opportunity to expand the focus on primary health care, mental health and social work type functions within paramedicine. But of course there needs to continue to be a focus on those high acuity, low occurrence events that happen across paramedicine, and that paramedics are still required to respond to. – *26, Paramedicine, Executive*

I think it's a really necessary part of the puzzle. But the barriers here to it, currently, I'd say is a trusted educational program. – *19, Medicine (ED), Policymaker (State)*

[We need to be] understanding it from those that have expert knowledge. – *51, Paramedicine, Clinician-Researcher*

You do not need to be an intensive care paramedic to specialize in primary or urgent care. – *5, Chief Officer (State)*

Paramedics and a GP's job is knowing how to break the guidelines safely, because the guidelines apply to a very, very narrow group of people. – *64, Medicine (ED), Policy (National)*

If you don't use a skill set, you lose it. – *14, Paramedicine, Academic*

Master's degree is mandatory, I think. – *52, Paramedicine, Policy (National)*

I'm not sure that we need to go where some of them want to take it, which is to being a Master's degree to be a community paramedic. – *7, Paramedicine, Clinician*

The skill we need is the skill of patient assessment. And if we can do a thorough patient assessment and we can make really solid decisions around what we're seeing those patients will have a better outcome. – *81, Paramedicine, Manager*

At the moment it's a four week course pretending to be a Master's degree. It's safe and it really works, but it's very vocational. It absolutely piggybacks off the carefully selected competency of an advanced ICP, their pharmacology and pathophysiology and the autonomous 'can do' spirit. – *1, Paramedicine, Manager*

When I was considering primary care paramedicine, was, how do I get that extra qualification? And when I looked different, universities are offering different things, and there was no clear job. So it's like, well, how am I going to spend 3 years and tens of thousands of dollars on something that might not credential me for the actual job at the end. And that's why I've sort of sat back. – *51, Paramedicine, Clinician-Researcher*

[Referring to ICPs as CPs] I think it'd be difficult actually to keep both. But I don't think that you should be precluded. – *5, Chief Officer (State)*

I'm not being disrespectful when I say some of the courses that are out there, I've looked at those courses, but they don't give me what I need to have. – *81, Paramedicine, Manager*

Some services, I believe, use intensive care paramedics in the primary care role. To me, you couldn't get to two ends of the spectrum further apart. Yes, those people have got good critical thinking, but they tend to be sort of resuscitation focused very rapid thinking. This is a role where it's the Type 2 thinking. You need to sit back. You need to start to analyse the patient and really look at this big holistic situation. – *51, Paramedicine, Clinician-Researcher*

When I phone up a GP I'm more articulated. The training we've had aligns with GP training. I can articulate what I've done in language that a GP understands. – *51, Paramedicine, Clinician-Researcher*

Take your average GP and stand them next to your average ED physician and you're going to see two very different characters, two different ways of thinking. – *51, Paramedicine, Clinician-Researcher*

If I was God and ruled the world, I think we would have a national curriculum. – *51, Paramedicine, Clinician-Researcher*

Use of a rotational model

I think there's an absolute value for paramedics doing 50% in urgent care, 50% on the road because you keep those skills, and you keep the primary care. – *67, Paramedicine, Manager*

It's actually exceptional. And if you were looking at a practitioner model, it's really, really good, I must say, in that it continues to support the development of currency. We don't have anything even remotely close to that in the Australian context. – *5, Chief Officer (State)*

I love the rotational model. I love the idea of a paramedic being attached to my GP clinic, but I see costs for that. I don't know who'd pay for it, so I don't see that happening. – *99, Medical (GP), Clinician*

I think that those models would be great. – *41, Medicine (GP), Policy (State)*

Can learn so much from GPs and ED by working in those sort of rotations. Fantastic. I think that hands down. If I could rule the world as a primary care paramedic: some time in GP, sometime in ambulance and sometime in ED. – *51, Paramedicine, Clinician-Researcher*

Where I actually learned about primary health care was in a general practitioner's office, so actually had feedback. I had a room on my own, would treat patients, and the doctor would come in at the end of the consult. Mine would last twenty minutes, whereas the doctors last 3 or 4. But yeah, the doctor would come in the room and say, "What have you got?" and I'd summarise the history and I'd recommend some scripts, or some pathology, or some radiology of some kind and then the doctor, and I would have some dialogue, usually with the patient, sometimes without, about my skills and insights about primary health care. So I think that active engagement, not just within an ambulance service, but within the broader health care system is really important. – *52, Paramedicine, Policy (National)*

That would have been awesome just from that professional development perspective. – *6, Paramedicine, Academic*

Is this good for the professional development of ECPs and the clinical quality and patient safety? I suspect the answer to that is a no brainer in terms of rotating through different models of care in terms of that sort of fast track, ED type setting, and then a general practice type setting. – *26, Paramedicine, Executive*

Placement, so paramedics in emergency departments, paramedics in GPs, in different aspects of the health service, where we're likely to need to integrate and collaborate. – *51, Paramedicine, Clinician-Researcher*

It's a whole new nuanced area of care, and they need to be actively engaged in the broader health care system, not just an ambulance service. – 52, Paramedicine, Policy (National)

No one size fits all: there must be local variations

Every community will be absolutely different. – *5, Chief Officer (State)*

I really don't think that there would necessarily be a one fit for all solutions across all the state and territories. – *75, Medicine (ED), Policy (State)*

This isn't one cohort. This is lots and lots and lots and lots of little different cohorts, and we need to identify those needs. Different models that suit the region of response which ties in with ambulance service. – *51, Paramedicine, Clinician-Researcher*

We go into an area and tell them what we're going to do - that doesn't work. What we need to do is put people in the ground in the area and find out what is missing from that area and then target the services to match the needs. No point in reinventing the wheel, no point in going to somewhere where you're not needed. So the projects I've all been involved in have gone in, and the first 6 months is just spent finding what's needed and then targeting our services to match what's needed, and that's why all of our community paramedic roles are different within the State. – *7, Paramedicine, Clinician*

We should probably steer away from having a one size fits all approach. – *2, Paramedicine (Intensive Care), Clinician*

Once we understand what the community needs, then we have a look at what those jobs are that the ambulance is already attending there. – *81, Paramedicine, Manager*

It's really superficially appealing to try and do this the same in every single bit of Australia. But we all know there's vastly different resources, cultures, networks, etc. – *64, Medicine (ED), Policy (National)*

We need to actually go in and understand the community before we start the program. – *81, Paramedicine, Manager*

It's not a one size fits all system. – *1, Paramedicine, Manager*

Setting the principles, allowing people to do things slightly differently. – *64, Medicine (ED), Policy (National)*

The main thing that I've observed that really makes it fall over or succeed is A) how well they're integrated into the community, and then B) what pathways they can offer to patients. – *80, Paramedicine, Manager , Paramedicine, Executive Director*

I think it's probably highly variable, depending on the different communities we have. – *26, Paramedicine, Executive*

I'm still in really two minds about the place where ECPs actually fit into the health care system compared to CPs. The key word that I like to go to is augment, it is really there to augment the existing health care system. This is really about community and about really finding out what are the gaps in that particular community at that particular time, and are ECPs or CPs the best paramedics to do that job? – *6, Paramedicine, Academic*

I mean, some people will say, "Oh, you need a national approach". I'm not sure whether that's the case. – *13, Government, Policymaker (State)*

There was a big GP super clinic, like urgent care clinic, that opened up 24 hours, and we had a look, and we actually saw that it only reduced our hospital ED presentations by 1.4 patients per 5 days. Yeah. So, yeah. – *75, Medicine (ED), Policy (State)*
Do you think, then, that it wasn't the right solution for the right place? – *14, Paramedicine, Academic*
Yeah! That's the thing I'm trying to say. It has to be tailor made. – *75, Medicine (ED), Policy (State)*

If you're a lady with an uncomplicated urinary tract infection – which really should be seen by a GP, so we say ED avoidance but with GP replacement – there are no less than five state funded agencies that can go and treat that lady somewhere other than the ED. So there's this multiplication of resources. – *1, Paramedicine, Manager*

What are the types of jobs that we go to in those communities and then how do those jobs then look

against the national data? So do we have a sudden increase in one area of rheumatoid arthritis and autoimmune disease and what's possibly causing that, and what health literacy is out there? – *81, Paramedicine, Manager*

The other thing that I would like to see in a really good policy is stakeholder engagement, lots of engagement with various colleges, governments, research groups, universities and the public. – *91, Health workforce analyst (State)*

Our predominant experience is that there are opportunities for these models to work well when they are properly integrated into a broader primary health care system, and indeed, within the general public system, as well. So critical to the success for us, has been access to urgent care centres, access to various community nursing, wound care and other programs and access to GPs that we can refer patients through or access medical appointments for if a patient doesn't need transport. – *26, Paramedicine, Executive*

Don't go it alone: building relationships with local primary care providers and patients

Dropping them out of the loop, that's no good for their service. That just sees us stealing patients from them rather than collaborating with them. We work in a collaboration with GPs. – *7, Paramedicine, Clinician*

Co-design, co-design, co-design. – *70, Patient*

What you're wanting to do is upskill the emergency department about what the paramedics' skills are, and the GPs as well. It's all about relationship building really, at the end of the day. To be able to build a functional team, you all need to understand each other's skill sets, and what can and can't be done in each of those settings. And given that the paramedic is often that meat in the sandwich, build the confidence in both the GPs and the emergency department that the skill set of the paramedic is actually going to do that correctly. – *88, Medical (GP), Policy (State)*

I know every single pharmacy that's open late hours, which GPs will take a favour from me. It's very, very individual dependent, currently all those referral pathways. – *64, Medicine (ED), Policy (National)*

I walk into our local medical centre and catch up with them, see how people are going. Refer in. They refer out, type thing. So, when I started talking to them I noticed that they started, you know, picking my brain about patients. And the same I would go in and go, "Hey? I saw something yesterday, and I didn't really understand what it was, but they've been diagnosed with blah, blah, blah!" And then they would help me. – *81, Paramedicine, Manager*

I'm really, more often than not, the eyes and ears for the GPs. I can come up with a care plan, and I'll discuss it with them, and they'll either give me a tick of approval, or they'll say, 'no, I want you to do this instead.' And it varies from doctor to doctor, you know. – *7, Paramedicine, Clinician*

The communication job there is enormous like, I need to know not only what fits into the respiratory, what fits into cardiology, what fits into the chronic wound clinic, but also how to navigate the communications around it, and that's similar in general practice, in my very limited experience in general practice environments and that's not easily replaced with just protocols. – *64, Medicine (ED), Policy (National)*

As a GP we all have to do emergency department stuff. So I know what happens in the emergency department, and in my rural terms, and some of my other things I've had to go out with the paramedics and do paramedic stuff. So I sort of feel like I know what it's like. To me it is more about that actual

relationship building of a team. – *88, Medical (GP), Policy (State)*

I would love to suggest that you have co-design. You need to have people like ourselves working with paramedics and working with the government if you're going to introduce a new system. Have our voices. Hear our experiences. Test out new ideas so we can tell you, yes this feels comfortable, no this does not feel comfortable. – *70, Patient*

I see ambulance services playing in the area and not really building up networks. – *21, Paramedicine, Academic*

There needs to be buy in from the other people who are doing that work. – *91, Health workforce analyst (State)*

It's really important that the people have a good understanding of the local services available. – 48, Paramedicine, Clinician-Academic

You would need not only institutional buy in, but buy in up and down the chain in the departments of health, and those folks that we partner with or who's professional boundaries we would encroach on. – *20, Paramedicine, Academic*

That's what's been lacking in paramedics working in the primary care area, is longitudinal care as a transaction between providers. – *21, Paramedicine, Academic*

There is a role to play for reactive models, but they work really well once they're integrated. Ambulance services as truly integrated within health care services, not in a standalone silo on their own. I think that's key. – *95, Paramedicine, Academic*

We don't want to be replicating services that are actually already there, because that would be very inefficient use of the health service. Similarly, though, can we deal with somebody's problem and avoid them accessing another more expensive part of the health care system? – *51, Paramedicine, Clinician-Researcher*

Access to on demand specialist consultation

I think it's good to have the shared model of care because it's not just one person making that decision. As ambos we typically have a shared model of care, because we have an offsider that we will bounce it off. I don't have that luxury. So it's always good to have a second set of eyes, and it's always good to make sure what we're doing is on the right path. – *7, Paramedicine, Clinician*

Anything with telehealth. As long as you have access to a supervisor, so that they don't work outside their scope practice, anything like that is very safe, and has been very well proven. – *25, Medicine (ED), Clinician*

Instead of having the physician on the phone as an advisor that could be a consulting paramedic who knows the environment you're working in and has learnt those skills. What's wrong with that model? – *51, Paramedicine, Clinician-Researcher*

Consultation would be very useful. So we tried to have an arrangement with the local hospital whereby we could contact the SMO in fast track, for example, to sort of go, "Look is this something you would need to see or do you feel this is better suited elsewhere?" So informally we've built relationships with GPs, consultants and even now, with the ambulance service there is now access to higher level consultation. So there's physicians, doctors working in the clinical hub. But again they need to be available

sort of on the same hours that we work. You phone up there's not a doctor there. – *51, Paramedicine, Clinician-Researcher*

I think when you get these low acuity jobs, they've got to have clinician oversight. I think that's what makes it work well, is it's got to be passed to clinicians to then sort out whether it's genuinely something that could result in a non-transfer. – *9, Paramedicine, Clinician*

I think the idea that paramedics must consult, or need to consult with a physician in order to initiate care, I think, is a little overblown. That's not to say that safety netting couldn't add value, it adds value. I don't think it's absolutely essential. – *26, Paramedicine, Executive*

Do we need to educate our paramedics rather than have them just phone a doctor? – *51, Paramedicine, Clinician-Researcher*

I really like the idea that the paramedic can be supported remotely with, if they're doing primary care - whatever you're gonna call it - kind of work to actually have a GP that they can co-consult with, or whatever for some things. Some things won't require it. Some things definitely will. And I think that that would make it a much more satisfying and safe and enjoyable thing for the paramedic involved to be able to have that support. – *41, Medicine (GP), Policy (State)*

I think you've got to have a mix of brains. – *9, Paramedicine, Clinician*

Access to specialists, so it could be midwives, it could be mental health, could be social care. So, having a range of experts that we can refer to, to support our decision making, making joint decisions about somebody's care with somebody that can provide that level of input as you would in hospital. – *51, Paramedicine, Clinician-Researcher*

You're going to need to potentially talk to a wound care CNC. So you've gotta have a network of people that you can get a hold of that's gonna help you make a decision about something like this. – *9, Paramedicine, Clinician*

I think paramedics work in the primary health care space, or are starting to work in that primary health care space, need a framework around them that makes them part of the multi-disciplinary team and I see that multi-disciplinary team being physician led. – *21, Paramedicine, Academic*

[Consultation] works really, really well for that group that's between the practitioner and the general paramedic workforce. You're a specialist. You have specialty schools, education, training. You have access to some different assessment tools. You may carry some different medicines. You're still working under your employer, and you're still working to a set of clinical practice guidelines, but the telehealth component allows you to better connect and refer those individuals into primary health care. It also allows you to use the skills from whomever it is you are conferencing with to extend beyond your clinical practice guidelines and support the delivery of the care that the person needs in the community. – *5, Chief Officer (State)*

You need that line of clinical support, and then that also backs up your paramedic… you know, if it comes down to who is standing up in front of the coroner, you had that line of accountability, that line of backup. You're backed up. – *99, Medical (GP), Clinician*

[When asked who should be available to consult] I think there are GPs who have cred in the Emergency Physician's eyes, and GPs who don't. – *99, Medical (GP), Clinician*

[When asked who should be available to consult] I think my bias with it being a GP is that GPs would always err on the side of caution, and probably send them to the ED. Whereas if I just was doing a shift where I gave phone advice, I'm pretty comfortable with that. If the question is, does this person need to go to the ED or not, the person who runs the ED is probably the best person to answer that. – *25, Medicine (ED), Clinician*

Staffed by perhaps an ED physician and a number of GPs with special interest, because it's more economical and affordable a workforce. And that covers for your tricky ones where that GP might want to refer to their ED colleague. – *99, Medical (GP), Clinician*

The most flamboyant example I can think of is where we had a dementia patient who had somehow managed to tear his nail back from the base… and there was bone sticking out, and nail going everywhere, and like it's effectively a traumatic amputation. There's a fairly clear intent from the family he's not going to hospital, but you just can't leave the stump of a finger there. We spoke to [a consultant physician] and he said, have you got a rongeur?... It's a bone nibbler, and you just crunch the end off the bone sufficiently back that you can create a skin flap to go back up over the end of the finger and you have effectively closed a traumatic amputation. You are never going to find a guideline for that… [Consultant physician] is so bloody country he said, 'Look a pair of shears will probably do it.' – *1, Paramedicine, Manager*

[When asked who should be available to consult] If you did have GPs operating these services, you'd want to have ones who have done some training, experience in this area, but also making sure the GP who is running this is supported by the ED doctor. – *99, Medical (GP), Clinician*

[When asked who should be available to consult] You'll need to staff with GPs, there's not enough ED doctors to go around. But if those GPs are employed as special interest – GPs with a specific interest is the new acronym, GPWSI – you'd have a really good workforce, and there's a lot of those. Who work in EDs, who could help support this program. So yeah, I want it yesterday. – *99, Medical (GP), Clinician*

We have a set of guidelines-protocols-rules, and then we have everything else. The way in which we structured ours is to take the senior ICPs, so they've got that autonomy, they've got that confidence. 'I don't have a guideline for this, I maybe don't even have the drug or the tool, but I reckon I know where I could find it.' So they'll call [a specialist physician] and say, 'Here's the problem, here's a picture of it or some numbers, I reckon what I need to do is this, what do you reckon?' – *1, Paramedicine, Manager*

[The consultant physician] has got to be really ambulance fluent, but also I think like a country GP-surgeon, sort of like that rural generalist 'can do' spirit… [the physicians] say, 'If it doesn't absolutely need to be in hospital give me a yell.' – *1, Paramedicine, Manager*

You've got to be supported. You've got to feel like you're not being hung out to dry politically or clinically, when you're going out to do work that is inherently, really very difficult. These paramedics will be seeing probably some of the most difficult patients in in the community. – *41, Medicine (GP), Policy (State)*

Referral pathways

Paramedics, I don't think, refer very well to general practice. I think that they tell people to go and see their doctor. I'm not sure that's a referral to general practice. I think it's a cop out to write on a case sheet. Doesn't help the patient. Doesn't help the paramedic. – *5, Chief Officer (State)*

You can refer them to the physiotherapist, occupational therapist, or whatever it might be, or the nurse, you know, the particularly the community nurse, to deliver the services. And you have a system set up where they can get something in a timely manner, like within 24 hours. – *3, Chief Officer (State)*

It’s not just informal relationships. It’s formal referral pathways as well. – *26, Paramedicine, Executive*

There is no referral pathways for people. And that's an enormous failing. – *4, Paramedicine, Academic*

The bigger piece of the pie is actually having an electronic referral process system. – *95, Paramedicine, Academic*

Physio is probably the most likely one followed by community pharmacy. And if we discount the capacity of our community pharmacies then we really haven’t been paying attention to the evolution of that space in the last 10 years. So there’s many places we can send people. – *5, Chief Officer (State)*

You must increase capacity. So increase the corridor. Widen. You must increase the access to alternative care providers. – *95, Paramedicine, Academic*

Paramedics don’t have a current referral pathway into Allied Health, and that absolutely needs to change – *5, Chief Officer (State)*

In terms of Allied Health, absolutely. We should be using Allied Health and steering people away from even general practice. That way there’ll be more general practice spaces for people that need general practice. So without question. You know, dietetics, maybe not as commonly. But if you are a specialist paramedic in a primary urgent care, then you might. – *5, Chief Officer (State)*

I keep hearing people can’t find a GP. They can’t access a GP. So we actually need to proactively design a system where everybody is fed into a GP and we have, you know, you guys have got a good relationship to be able to say “We’ve got Mr. X. He really needs a GP. Can we work with you for you to take on his care?” – *88, Medical (GP), Policy (State)*

Every time you send an ambulance to local football and netball club for the one broken ankle, you end up being asked to deal with 3 different soft tissue injuries while you’re there. They should be referred to the physio and not trouble general practice and definitely not trouble the emergency department. – *5, Chief Officer (State)*

If we were to ring fence the paramedic role to purely resuscitation and transport, the key thing is that for the non-emergency stuff, they would need to have pathways in place. So, if it’s, “No. Paramedics shouldn’t do any kind of treatment other than resus. They should refer.” Okay, but refer to where? – *63, Government, Policymaker (State)*

A child with osteogenesis imperfecta who sustains a soft tissue trauma that’s likely to have an underlying fracture, sending that to a primary care centre is just nuts. It only delays their arrival ultimately where they’re going to end up, and it’s going to be a complex orthopaedics clinic and they’re likely going to need surgery, because they’ve probably got an underlying fracture that’s not going to heal. So making good decisions at the beginning can actually shorten their time and actually reduce the cost. – *5, Chief Officer (State)*

Unique models, funding, and performance indicators for rural and remote services

Paramedics in rural areas and underutilised… some remote stations might do 2-3 jobs are week at best. We could get a lot from that paramedic doing preventative primary health care within that community. – *2, Paramedicine (Intensive Care), Clinician*

The sheer volume of workload in a metro area means that your cost benefit analysis always looks better in a metro area. Your cost benefit analysis is always going to look shithouse in rural areas. There’s no two

ways about that. – *9, Paramedicine, Clinician*

I can see benefit in large urban areas where you have high population density, and you know hundreds of thousands, if not millions, of people, within a reasonably contained geographic area… I recognise that a lot of the benefits in rural locations are not particularly economical in many respects, but they provide a health care safety net where there would otherwise be nothing. – *26, Paramedicine, Executive*

This is the difficulty that's faced every State Government since the beginning of time is, how do you justify the expense of all of this in a rural area where you've got, you know, a population of 1,500 people. I don't know the answer to that. – *9, Paramedicine, Clinician*

There's a real lack of resources in the country. Like, I'm thinking about the places that are often run by like remote area nurses, and they might not have a GP for like, weeks. Or there's one GP who's on call for the whole town, and they can't seem to get anyone to replace them or cover them, and they're usually very exhausted, and they've also got their own workload in their clinic during the day. So, anyone who can take the after-hours onus off our GPs in country centres would be very beneficial, I think. – *25, Medicine (ED), Clinician*

Acknowledgment that rural is very different from semi regional to city, that there are certainly very different needs. For example, for me a decision might be based on, "Well look, a GP or a hospital is actually only 20 min away". Whereas somebody regional, that's a 4 hour trip, and even aeroplanes involved. So the rationale behind my decision making would be very different if I was placed in those regions. – *51, Paramedicine, Clinician-Researcher*

Paramedic models, the care pretty much are based around big cities. Not going to work quite in the same way in the bush, and they're really not going to work all in remote. – *66, Patient Representative*

Multipurpose services, out rural. There's no doctor there, there's usually just an RN, a couple of ENs, a couple of AINs, half the facility has been turned into a residential aged care and it's very difficult to find nurses that enjoy working in the emergency department side of that. – *9, Paramedicine, Clinician*

The ambulances and services have been set up with state-of-the-art audio visual. So if someone's doing an, I don't know, a 300 km run and there are half a dozen people they saw the week before, why can't we book in a whole lot of teleconferences. One's chatting to people while the other one's driving, and they swap around, and you get value that way too. – *66, Patient Representative*

I've never worked metro, but in rural communities. The further you get away from a service centre, the less services that are available. – *81, Paramedicine, Manager*

I think it would be easy to get the buy in in rural areas, because there are very few doctors. – *91, Health workforce analyst (State)*

In rural areas the follow up probably is going to end up being a paramedic, and probably needs to be in some ways. But that's not done well, and there's not really the infrastructure, I don't think, at the moment in any of the programs to do that. And we know that the follow up helps to reduce repeat, you know, hospital admissions and that sort of thing. – *48, Paramedicine, Clinician-Academic*

We can definitely see opportunities for paramedics to be working in primary care clinics in remote parts of the State, where general practitioners or nurse practitioners are not present, and have not been able to be recruited to. – *26, Paramedicine, Executive*

I think outreach would be a really good idea for disadvantaged populations. But I'm not sure. I think if

ambulance tries to do outreach you'll spread yourself too thin, and then none of your programs will have a chance of being successful. – *41, Medicine (GP), Policy (State)*

Viewpoints on specialist paramedics as part of multidisciplinary teams

Paramedics joining multidisciplinary teams, I think they are really well placed to do that because we already know that paramedics are called to aged care, or even health clinics, when the acute issue of a patient goes above and beyond the scope of the nurse or the GP, who doesn't really work in the acute space that much. You know, they did their placement 10 years ago or whatever. So I think that a nurse skill set, a paramedic skill set, a GP skill set, are complementary. – *85, Paramedicine, Policymaker (National)*

If we were to see paramedics joining the multidisciplinary team in providing health care, primary health care, to the community. That's a really great idea. – *30, Chief Officer (National)*

I actually don't think in an emergency, urgent care setting, I don't think that involving everybody is actually what you need to do. That's the next steps down the line. – *88, Medical (GP), Policy (State)*

There are models around, and there there's dozens of them, both in Australia and elsewhere where you do have usually a multi-disciplinary team that can see someone within that 12 hour period to actually assess. This is where, rather than still sending out a car, you're sending out the right people to provide that care. – *3, Chief Officer (State)*

Ensuring smooth transitions of care

Transitions of care I like to think of as the most dangerous times in care, because that's when information gets lost, people make assumptions. No one checks. Something's not there. What mechanisms allow a safe and smooth transition in terms of quality and safety? – *66, Patient Representative*

That paramedic still does not provide a continuity of care. That paramedic just provides one episode, possibly two, and delivers them to a much more expensive setting, if they take them to hospital. – *4, Paramedicine, Academic*

Good governance and long-term investment

[Governance] So obviously, first, it's the governance. There needs to be really strong governance, guidelines and protocols. – *75, Medicine (ED), Policy (State)*

For this model to work there's a lot of organisations, government things that need to get on board with it… If they're not on board this is never going to go anywhere. It's important because it has an impact on scope of practise, it has an impact on accreditation processes, it has an impact on training requirements, and funding mechanisms for how we going to pay for this. – *72, Paramedicine, Academic*

It takes a bit of government will, and policy changes. – *45, Parliamentarian*

What would make roles like this work is good governance, but of course I would say that because that's where I work, you know, good guidelines, good quality assurance. But good clinical governance to me is not just good guidelines, good data, good rules, etc. It's also good networking, good integration, good health information systems. – *65, Paramedicine, Manager*

[Long-term investment] This trial approach to a thing often means that, 'Oh, there's only been a 1% improvement in this. It's not worth it changing everything for that.' But actually in year ten, there's a 15% improvement, and that's thousands of presentations, and that makes a massive difference. – 85, Paramedicine, Policymaker (National)

A system where the paramedics are valued and highly trained. This should be our best people, and this should be seen as this Paramedic Practitioner pathway is at a similar footing to an ICP. – *2, Paramedicine (Intensive Care), Clinician*

It takes time to embed models. – *85, Paramedicine, Policymaker (National)*

How do you actually get around that legislation? Bigger things like prescribing. – *55, Medicine (Emergency) Policy & Clinician*

I think the government, instead of creating larger hospitals with no one to staff them, should focus more funding here. – *85, Nursing (Emergency), Clinician*

Managers and policymakers aren't interested

The whole clinical system is run by, you know, and influenced by one person, not by a group of people who have a really good overview of the health care system. – *4, Paramedicine, Academic*

[Commenting on the manager's perspective] It's not solution focussed. It's just accepting that there's a problem and not providing any other alternatives. – *48, Paramedicine, Clinician-Academic*

There's no long term planning, you know, and all our planning is based on political cycles, unfortunately. And that's, I think, where we are failing. It goes on a political cycle. – *4, Paramedicine, Academic*

What government's going to do that with 3 year and 4 year political cycles? It's not going to happen because as soon as something goes wrong the media, the opposition will hammer it home, and the public will jump on board and hammer it home, and you don't get your opportunity to stay in office, and the ones that have criticized you get into office, and they have to tweak it because they can't continue it on, even if it's a good system, because they can't be seen to be using the same system. You know it's crazy. – *62, Parliamentarian*

[Commenting on why there is no competitive need for change] Jurisdictional ambulance services have a monopoly. – *4, Paramedicine, Academic*

Buy in from the actual organizations themselves. Like, I think our ECPs work really well, and the work they do is really good but it also depends on what sector within [the state] you're in, because some managers have a high opinion of ECP, some managers don't see their worth. – *49, Paramedicine, Manager*

Inappropriate dispatch of specialist paramedics

There's no question that the ECP model falls over in [service] all the time, because they get used as clock stoppers, because they become the only resource available. So they get sent to the 1As and the 1Bs, because there's no one else. And so their work is so often subsumed by operational demand. – *9, Paramedicine, Clinician*

Seriously? Like, oh, we got there so quickly, well done. Tick. You pressed your buttons quick enough, and you arrived at the person who has a sprained ankle within 25 minutes that really could have waited 25 hours with some advice on the phone, with some RICE and compression and a physio referral. Great, good job! Tick. Is that what we want for health care? I don't think so. We want equity and accessibility to health care. – *48, Paramedicine, Clinician-Academic*

Dispatch. We've been working for eight years, there's still, within our organization, the people dispatching, we've been having to sort of educate that from the ground up. That's a big frustration. If we're not being sent to the right job we're tied up, then we have to call an ambulance, so it's very inefficient. – 51, Paramedicine, Clinician-Researcher

We're wedded to response times. For what reason? – *62, Parliamentarian*

I do also like the idea of them not having the ability to go code one. As soon as the operations centre know, they will use them for code ones. – *2, Paramedicine (Intensive Care), Clinician*

This is what it should be used for, and nothing else. And them being free to do what they're doing. – *49, Paramedicine, Manager*

If you're mandated by the Ambulance Service Act, which I think is from 1986, or something like that, it's about getting to the highest acuity of patients in the shortest amount of time. And if that's what your KPIs are driving you towards, that's all you care about, and everything that you do around low acuity work is literally just to remove them as a "problem", so that you can actually do what you're being assessed against. So that fundamentally needs to shift, I think, moving forward. – *95, Paramedicine, Academic*

There is still an extremely strong focus on response performance, despite there being no correlation to outcomes. – *26, Paramedicine, Executive*

Other managers will take ECPs off ECP vehicles and split them up onto general duty paramedic cars, because then their view is, they've now got 4 ECPs running around who can use their ECP skills, despite not having the equipment that's in the ECP cars to do it. – *49, Paramedicine, Manager*

The clinicians in there are a combination of registered nurses, and then very senior, you know ambulance clinicians. So, ICP, ECPs and there's some doctors as well, they've got access to physician advice too. And so hey keep an eye on the board. – *9, Paramedicine, Clinician*

I think what works well is their ability to look at the whole board. The ECP model tends to be, one person on the board has already run this job through an algorithm and gone, this is ECP appropriate, we're sending it to the ECPs. And that's not always terribly effective, I don't think, whereas the [multidisciplinary] approach to triage is much more wide ranging. – *9, Paramedicine, Clinician*

It makes it difficult for people to know what to refer to us… Having an ECP up in our call centre [is essential]… on the dispatch side of things, having someone who is trained up doing the dispatching, rather than having general dispatchers. – *1, Paramedicine, Manager*

Often the ECP gets there and they go, 'yeah, this person's got to be transported. I'm now going to sit here for two and a half hours and wait for an ambulance to turn up so that they can be taken to hospital.' And actually, if a clinician had made a call back in the first place they would have already been able to nut that out. – *9, Paramedicine, Clinician*

Operational demand is always going to supersede everything else. – *9, Paramedicine, Clinician*

A lack of national practice standards for specialist paramedics

You've got a practice expectation from the Nursing and Midwifery Board around nurse practitioners and the Paramedicine Board needs to create the same, so that there is clarity around what you should be able to expect. We need practice standards to be set, and the best place for that to occur would be from the Paramedicine Board. – *5, Chief Officer (State)*

We do need to be really clear around where their scope starts and finishes, and that we have a framework that supports that. – *13, Government, Policymaker (State)*

[What we need] to make it work is appropriate credentialing. – *51, Paramedicine, Clinician-Researcher*

I have seen this language used a little bit to curtail the professional expansion of paramedicine. 'Oh, well, we don't know what they can do. They don't have national standards. They don't have 'X'. They don't have 'Y'.' And the reality is, we see variance in clinical confidence across all health professional disciplines, including medicine. Yes, we want to ensure practice is as safe as possible, though I don't think that is absolutely contingent on medical oversight, and I don't think that nationally agreed clinical standards are the panacea for all things paramedic practice. – *26, Paramedicine, Executive*

Ambulance services across Australia operate quite differently. – *91, Health workforce analyst (State)*

[Comparing to Nurse Practitioners] There's a pathway, and you ask a doctor what a nurse practitioner does, hopefully most of them would sort of know roughly that scope and limitations of practice. – *51, Paramedicine, Clinician-Researcher*

I think a lot of success and failures with these kind of models is defining what the remit of these clinicians is. – *41, Medicine (GP), Policy (State)*

The PBA isn't going to set what that is. They're going to let you do whatever it is you're prepared to, you know, risk manage from an employer point of view. – *5, Chief Officer (State)*

I definitely think the industrial relations landscape in paramedicine has some evolving to do. Which is not intended to be a veiled swipe, I should add. It's the maturation of the profession more broadly. Clearly, consistent national standards are important. – *26, Paramedicine, Executive*

A national standard so you could go, "I'm an "X" paramedic". The doctor goes, "Okay, they're capable of X, Y, Z." – *51, Paramedicine, Clinician-Researcher*

Who's going to provide the credentialing? Because we don't have any mechanisms available to us yet for that credentialing. – *21, Paramedicine, Academic*

It's very nice to have a defined scope of practice for an emergency department… a defined scope of practice and credentialing to it, is really nice. – 41, Medicine (GP), Policy (State)

Inconsistent terminology causing confusion

We have some language which needs to be firmed up. Very, very keen to see that done by Ahpra, the Paramedicine Board, so that it's national, and it is uniform, and it is absolutely consistent. – *5, Chief Officer (State)*

Nomenclature, it's all a bit confusing across the jurisdictions. – *13, Government, Policymaker (State)*

Like they are for every other term in emergency ambulance services around Australia, it will be unique in that particular jurisdiction, because Heaven forbid we actually all, I don't know, use the same language. – *5, Chief Officer (State)*

What is the definition of primary care in this model? Because primary care is every single person from before they're born until they're dead in every single body system. – *41, Medicine (GP), Policy (State)*

[Defining primary care] I see it much more in that holistic way that the WHO defines it, and for me that doesn't quite match with the notion of a primary care paramedic. For me the primary care paramedic is

much more in the ED space which is immediate care. – *64, Medicine (ED), Policy (National)*

Professional silos

Paramedicine, I think, historically, it's been very, very excluded from the rest of the health system. And I think, as a result we're not very well understood. – *51, Paramedicine, Clinician-Researcher*

I mean, look at the UK, they've got paramedics working in hospital departments. We don't do that here. It is that siloed approach. – *68, Paramedicine, Academic*

Everything seems to be happening in isolation, and I think that's a problem. We need a system which is supported by multiple organizations, by, you know, your RACGP, by ACEM, by whatever. – *21, Paramedicine, Academic*

There is a lack of interprofessional understanding of what the roles are of a paramedic and a doctor and another allied health team members, which does make that integration, I suggest, more challenging than it would have to be, if both sides kind of understood each other's practices with a little bit more. – *6, Paramedicine, Academic*

A collaborative approach with ambulance services, healthcare facilities, GP clinics, specialist services, hospitals, and the government is needed. – *87, Nursing (Emergency), Clinician*

I'm not sure that the ambulance services have traditionally engaged very well with others. Whilst they're trusted by the community, they're not trusted by pretty much anyone they have any business dealings with. – *5, Chief Officer (State)*

A lack of understanding on both sides. A lack of understanding of by ambulance services and paramedics as to what an ED will do by or won't do by way of assessment; there's a lack of understanding from EDs as to the capacity of ambulance services. – *1, Paramedicine, Manager*

I don't think we're using the paramedic workforce to its full extent at the moment. – *68, Paramedicine, Academic*

I think we're not used to the full extent. I think we should be actually more of an instrumental cog in the world of the health system, and you know, in places such as the ED. – *68, Paramedicine, Academic*

In emergency medicine we're pretty inclusive, like as far as I'm concerned, if someone wants to come and work within the team and see patients and help us we're very welcoming on the hope. And I hope that's the vibe that most paramedics have about emergency physicians. – *64, Medicine (ED), Policy (National)*

Paramedics do have capabilities to deliver so much more than what is perceived, what doctors actually think we do. We've been described as specialist generalists. – *6, Paramedicine, Academic*

I feel like there is still a slightly antagonistic relationship between the hospitals and ambulance services and primary health care, and you all need to mellow. – *1, Paramedicine, Manager*

This is going to take decades, but start to sort of integrate more, and actually have that a genuinely integrated health system where paramedics are respected, included, and involved in how we actually work best. – *51, Paramedicine, Clinician-Researcher*

No access to medical records, no sharing of medical records

The fact that you guys can't access MyHealthRecord, and… I can't believe… I'm both surprised and not surprised by that… really shocks me in a way. We should do that for everyone, because you need to know what medications they're on, and you need to know if their kidneys are functioning or not. – *99, Medical (GP), Clinician*

Paramedics have no access to a patient's health record. Which is just astounding. You know, it's like… they literally… if someone's unconscious they are walking in completely blind. – *85, Paramedicine, Policymaker (National)*

I want paramedics to see my medical records! What if I'm unconscious? '000' is the most important time for anyone to see my records. That's ridiculous [that they can't access them]. – *10, Patient*

I go to my GP a week later and they have no idea that the paramedics came, that I was in hospital. I have to try and remember. I don't know about the names of things, the medications. I can't remember. They need to talk to each other, not put it on me. – *16, Patient*

I would have presumed that once somebody rings up the number you would have access to information. *– 70, Patient*

I am not aware that paramedics have no access to my medical records. I am comfortable with them accessing my medical history. – *53, Patient*

A lot of the time it's repetitive questions. – *8, Carer*

I don't think you can assume consent. I think that could get people into trouble. All sorts of privacy; we're living in a privacy manic age. – *54, Patient*

I probably would be in agreeance to assuming consent in terms of access to medical records… it's an emergency situation, right? Life's at risk… you probably need to have some sort of, break the glass, overriding protocol to be able to have access to the medical records when needed. – *94, Patient*

I feel for the paramedics, because they're working not enough information to deliver care expertly and safely. – *41, Medicine (GP), Policy (State)*

From here you could go to two hospitals. They both run separate systems, they don't cross fertilise. One of them can't look up the records from the other. They don't even share X rays, so you can't centrally look up X rays or path results. So the level of change that would be required to enable that, I think, would be phenomenal. If you actually did it, I think it would have so many other benefits. – *12, Patient*

The research that's been done, it's normally 90% higher kind of agreement with people who are caring for them accessing the health care records. They don't specify who it is that's looking after them, so I think you'd find most people would have no problem with it. – *66, Patient Representative*

I definitely think paramedics should have full access to medical records. For sure. For someone who is having a really bad emergency, it's very important to know if this is normal for them. – *57, Patient*

We don't get the whole picture. We don't get GP notes. We don't get blood tests that the patients had in the community. That would be fantastic. A recent blood test, for example, phenomenal, that would be awesome. – *51, Paramedicine, Clinician-Researcher*

Look. I think if we're actually serious about improving patient health care and decreasing costs to the health system, then we need to actually be willing to share records across all the systems. My patients

actually expect that. They don't understand why I don't get access. 'What do you mean you don't have a copy of that. The ambulance guy did it, took comprehensive notes. Why don't you have it?' It's like, 'Well! It's not my fault. It's just that it doesn't get shared with me.' – *88, Medical (GP), Policy (State)*

Health consumers have an expectation that at some point that their health record keeping is shared amongst the various craft groups. Yes there are privacy considerations needed amongst all the different schemes, legislation around the country. However, I think, sitting behind that is an actual health consumer expectation that, "Well, why wouldn't my GP have access to the discharge summary from the ED presentation on Friday night? Why when I go to ED, why does the person not have access to my GP's notes?" – *13, Government, Policymaker (State)*

A universal medical record might have told you. But you didn't have access to it... If they don't have access to a universal medical record, they really have to say, I don't know, I'm not psychic. – *33, Patient*

Information is gold. In any problem solving situation, without that information, you're going to struggle. So they've got to have access... There's no doubt in my mind you don't have access to enough information. – *33, Patient*

I wouldn't be comfortable with it... I don't think people trust paramedics. I mean we don't trust GPs; there's not a lot of reason to. – *33, Patient*

Why don't we have [access to medical records]? You know, trying to even get your own results after having a broken foot or something. It's so hard. You'd swear it was someone else's foot. – *33, Patient*

The double doing, the double talking, the double information-ing, because you're all discrete entities. You should be one entity, all working to a common goal. And you're not. – *33, Patient*

The double talking, the double doing, double double double! What's his, Henry Ford? Didn't he invent the assembly line? He'd be having a seizure. He'd think we're insane. – *33, Patient*

People who are in an emergency situation, I just don't think that they should be expected to remember everything in those sort of situations. They shouldn't be expected to be like, "Oh, yeah, I'm on this medication". Or, "I did this 20 years ago." I don't think they should, as patients, they shouldn't have that responsibility on them. I feel like as patients who are in emergency situation shouldn't have to have any responsibility for anything. – *57, Patient*

From experience, a barrier – fix it, and it's in an enabler – is access to hospital records. Without a doubt. – *67, Paramedicine, Manager , Paramedicine, Manager*

I think it's only logical to be honest... Even at a very basic level like allergies, you know, let alone the medications that she's on, any particularly quirky diagnoses or issues that she's got. She's permanently bradycardic – they could think she's having a heart attack if they don't know that. – *12, Patient*

Despite leaving ED with the discharge summary, 2 hours later haven't got that, so we end up treating it as a new circumstance. Health records is possibly one of the biggest enablers for paramedicine in urgent care. Just the transparency, just so we can see what's going on. – *67, Paramedicine, Manager*

I'm definitely with [other focus group participant] on the patient records and the history in the background. – *75, Medicine (ED), Policy (State)*

I had access to all the GP clinics' databases, and could put direct notes in directly for the GPs and get messages directly from the GPs... I've also got access to MyHealth record and I've got access to the Australian Immunisation registry... I think they're all equally as important. I can stitch their wound up but then I'll link them in with the doctor. Get an authorization for an ADT. I'm allowed to immunize with the doctor's order, so I can do that. But to close the loop off entirely, I can now log on to AIR and register to that

immunization… We can do it all in one. Close it off this case completely and move on to the next. – *7, Paramedicine, Clinician*

If they had access to [health records], it would totally change their vision to how they're going to treat and the decision they make. – *14, Paramedicine, Academic*

You have patients turn up to EDs. They get a GP letter. They don't see that GP. That letter goes nowhere. – *21, Paramedicine, Academic*

We can't do it when paramedicine or ambulance services are siloed, and I think that to me is a bit of the issue. I'm well for privacy. I really am. We unfortunately have to get data, so the right people can see the right data at the right time. – *14, Paramedicine, Academic*

If care was delivered by a paramedic in the person's home, one of the important things to think about would be how that care was recorded and how it could be shared with the multidisciplinary team. – *30, Chief Officer (National)*

The primary health system at the moment is quite fragmented, and there's not good information sharing between the acute system and primary care. – *30, Chief Officer (National)*

South Australia have a really easy way. They can log in and see discharge summaries, different patients. The State of Ontario in Canada does the same thing and NHS as well, with the ability to look at people's underlying health care records to see what services already exist - linking them in with it. – *95, Paramedicine, Academic*

It's difficult in that you're only getting a very small snapshot of the patient. – *95, Paramedicine, Academic*

We have a barrier of information sharing. So it's not easy integration of information one way or both ways or either way. – *19, Medicine (ED), Policymaker (State)*

From health, so downstream of paramedics, I definitely see that it would be great for information to be shared as it's gathered and passed in one direction. – *19, Medicine (ED), Policymaker (State)*

I think it would be helpful. For the paramedic on scene, absolutely and linked into general practice information as well. And so, even if they just had the information like their current medications and allergies, it would be a start. – *19, Medicine (ED), Policymaker (State)*

Oh, it'd be amazing. It'd be lovely if, you know, the information just was there on my screen as I opened [the medical record] and I just had to check it with the patient. – *19, Medicine (ED), Policymaker (State)*

[Paramedic access to medical records] I don't think you'd meet anybody out there who would say that it's entirely unacceptable. – *13, Government, Policymaker (State)*

It's very difficult to join up private and public. – *19, Medicine (ED), Policymaker (State)*

It can sort of give you a bit of insight into why we're not fixing this person's problem. I need to get to the root of why they're persistently calling the ambulance. – *51, Paramedicine, Clinician-Researcher*

They don't know if you're trigger happy, and you call a lot, or when you call it's deadly serious. You may or may not disclose information to them because you don't trust them, or you forget to tell them in a way that someone with whom you had a long history knows all those things about you. – *66, Patient Representative*

At the moment, MyHealthRecord doesn't allow event summaries from paramedics. – *66, Patient Representative*

Something that health professionals often forget is telling your story can be triggering, right? And your story may not be complete for all kinds of reasons, including getting interrupted. – *66, Patient Representative*

For our community, patients who are very unwell, it might be cardiac patients that have been thrombolised, sent to Melbourne. They return home with a lack of a plan of what they've got to do going forward. They're not connected. Their GP isn't aware. So, there's no connection between the ambulance event and their GP. – *81, Paramedicine, Manager*

It is a double edged sword if I'm honest, because while it is fantastic to be able to sort of look at somebody's history and gain a little bit more understanding about them, that can lead to some significant bias as well. So I think, when I do look at that, I'm very careful. – *51, Paramedicine, Clinician-Researcher*

The ability for all of your health providers to understand your history, how you got to be where you are, what you're currently taking in terms of medicine, what investigations you've had recently or in the past, in a reliable way allows everybody to start from a far more robust place and make better decisions. – *5, Chief Officer (State)*

Without a UPI, at least on a state level but probably nationally, it becomes really, really difficult to ensure that you're matching the health record correctly. The best alternative would be MyHealthRecord if it was robustly being added into. Paramedic access to MyHealthRecord is possible. It's not easy. It's definitely not simple. But paramedic access is possible. – *5, Chief Officer (State)*

I would love to know, as a GP, which of my patients are continually rocking up with level 5 stuff, because the thing is, hospitals are so overwhelmed, we don't get the letter 'til 3 months later. – *99, Medical (GP), Clinician*

I think paramedics should have access to MyHealthRecord. And it should be easier to access. Very difficult for me to access my own patients' MyHealthRecord from my own clinical software. It's just really painful. – *99, Medical (GP), Clinician*

I think that's probably something GPs should be educated more on, because we don't do that. We assume everyone knows what we've got. – *99, Medical (GP), Clinician*

And the integration piece also includes integration with medical care records as well. So it's not just the physical integration of someone into a system by being able to refer them to an appointment or take them to an outpatient clinic, it's actually about being able to integrate with existing digital medical records. – *26, Paramedicine, Executive*

I do think that communication back is important, and I think that's feedback we've got from the paramedics as well. – *14, Paramedicine, Academic*

Also, a lot of people may be in situations where they can't physically tell you what's happened, or they can't remember. They might be influenced by, like, you know sickness, illness, mental health, things like that where they can't actually remember things. Like, they don't want to tell you things that are very important to their health that can totally contribute to what's happening, or how you can treat them before hospital as well. So I think they should definitely be able to [access medical records]. – *57, Patient*

I was at a meeting last night where they've developed a system where they are planning to roll out, at the end of this year, access for the GPs to see the hospital records, and the hospital will be able to see the GP records, which is huge in this country. The para staff need to be able to do it. – *88, Medical (GP), Policy (State)*

I would love out of this study for something to grow where GPs by region are given that data by the EDs. – *99, Medical (GP), Clinician*

Data that is unreliable or not shared inhibiting informed decision-making

ROGS data is terrible. If you take data which we know to be far more factually accurate, and if we map out what's reported in ROGS it comes nowhere near the numbers that are reported. So we objectively we know that there is clearly an error or a problem or a reporting challenge. And each jurisdiction does report their data into ROGS differently. They interpret the question differently. – *5, Chief Officer (State)*

The Report on Government Services, ROGS, it calls itself an experience survey. It's actually just a satisfaction survey. It's not validated. It's not psychometrically tested and interestingly, it's produced by the Council of Ambulance Authorities. Funnily enough, the same people that provide the service. So you can't tell me there's no bias there. That's used as a reportable government service output. How terrible is that? – *51, Paramedicine, Clinician-Researcher*

I don't think data quality should be an afterthought. It's the main issue. – *65, Paramedicine, Manager*

Looking beyond the report on government services data from the Productivity Commission, because it largely provides pretty ordinary data. – *26, Paramedicine, Executive*

There is so much more to be done in that national data set space, health economics and patient reported experience and outcome measures being another important measure. – *26, Paramedicine, Executive*

The ROGS data has really not kept up with the pace of secondary triage or community referral pathways. And indeed here's very little patient reported outcome measures or patient reported experience measures in that data. – *26, Paramedicine, Executive*

Data is impossible for us. – *64, Medicine (ED), Policy (National)*

I spend half my life churning through data that tells you very little trying to extract something from it, because we have so much data, and none of it really tells us anything. – *65, Paramedicine, Manager*

What would improve ROGS would be a standardized set of performance indicators that were established. The Australian Council on Healthcare Standards will release the first iteration of paramedicine performance standards later this year. And there's no obligation for people to follow those, but at least there will be a standard, and it'll define how you should calculate the answer. So, if we could get people reporting on the same metric, then it will become more reliable. – *5, Chief Officer (State)*

CAA could, given the fact that it's government services, make some recommendations. I think what they will do is, given their involvement in the ACHS, work is probably just try and get everyone to use the ACHS material. – *5, Chief Officer (State)*

We're so diagnosis fixated, which is driving a whole bunch of wastage and anxiety, and at the moment the data is all around diagnoses, and that just doesn't work. – *64, Medicine (ED), Policy (National)*

The main issue is actually data quality and the practical issues of collecting that data. – *65, Paramedicine, Manager*

We get some data out of the report on government services… That we don't use. – *4, Paramedicine, Academic*

Unless we have a strategic goal federally for health care, what is the point of measuring anything? Who cares? – *48, Paramedicine, Clinician-Academic*

The data is giving us false, unrealistic information. – *51, Paramedicine, Clinician-Researcher*

Data collection is key. – *95, Paramedicine, Academic*

These services can be a bit of canary in the coal mine. Consumers are ringing up with salmonella poisoning or COVID, or whatever it is. How was the data that comes from the services de identified and used to identify health trends and then alerts that go out to jurisdictions? I think often health doesn't use data as well as it could. That surveillance work can be incredibly powerful. – *66, Patient Representative*

Funding models that act as disincentives to non-transport

In some states in Australia the ambulance service is paid more to transfer. So there's a desire to transfer, because it's their funding model. – *85, Paramedicine, Policymaker (National)*

Funding is my biggest beef. They're paid to transport people to hospital. So why would you, as an ambulance service then go, “Hey? Let's like not take people hospital. Let's take them to where they need to be, or advise them appropriately”, when all the funding is based on transporting people to hospital. It doesn't make sense. – *48, Paramedicine, Clinician-Academic*

Funding of ambulance has traditionally been really quite weird, and in many jurisdictions it’s you still get paid ‘X’ amount of money, fixed rate, for attend with no transportation, and then you have a different remuneration, significantly higher, if you then transfer someone. The transport fee is 3 or 4 times the non-transport fee, which just is absurd in modern practice. – *5, Chief Officer (State)*

Transporting people to hospital generates more income. It also means that they’re less available to respond, and so they can service fewer patients. And so in some ways they get more money for taking you to the hospital, but then lose out because they can’t take 2 or 3 others to hospital at the same time. – *5, Chief Officer (State)*

We do continue to work very, very hard to steer people to better primary care. Then all of the emergency ambulance services will see a reduction in their annual income as a consequence of that if we don’t adjust the model in which they’re funded. And so a universal funding model which is used in Queensland would become necessary for all jurisdictions. – *5, Chief Officer (State)*

Funding disincentives that don’t support specialist paramedics

[After stating that they would like a paramedic to work in their GP clinic] We’re just so stymied by the narrowness of our funding system. – *41, Medicine (GP), Policy (State)*

The health care system can't afford it. It is not at the right price in any way, shape or form. – *3, Chief Officer (State)*

I have payment mechanisms on my list of big things that would need to be changed to make it work. – *65, Paramedicine, Manager*

The models that sit behind this aren't sustainable. And yes, it'd be great to drag paramedics into that space. But financially, they're not sustainable. – *21, Paramedicine, Academic*

Primary-urgent services that are available out-of-hours

I'd put the urgent care practitioner on at night. – *67, Paramedicine, Manager*

I would love it if we just had a GP service on site at 10 o'clock at night. – *25, Medicine (ED), Clinician*

I have a disabled child. I'm a single mom and I work part time, who has got caring responsibilities and often I find that the only time I can get the care I need for my child is during business hours. This means I have to take a day off work. I'm about to lose my job because I have to keep taking a day off work. In addition, my child misses a lot of school, because they have a health problem, and then they have to take a day of school to get a health care appointment is only available during 9 to 5. What would be better for our entire family would be if it was done on a weekend or after hours, so I wouldn't lose my job, they wouldn't lose time at school. – *66, Patient Representative*

The resources available 24-7 to avoid having to transport people in the hospital is what works. – *49, Paramedicine, Manager*

We still have outpatients that run from 9 to 12 and 2 to 5, etc. – *41, Medicine (GP), Policy (State)*

[Services need to be available when] it's the middle of the night. – *99, Medical (GP), Clinician*

There just isn't services available. So it's all well and good to say we're gonna send out this person who has additional training so you don't need to go to hospital. But on a public holiday, when there are no medical centres open, there are no X-ray places open. Someone with a minor fracture, for example, who could be back slabbed by the ECP, send for referral for an X-ray, and then follow up with their GP ends up going to hospital because there is no place to get an X-ray within the timeframe. Or a simple suture, when the ECP can't suture it, there is no other place other than a hospital open. – *49, Paramedicine, Manager*

I mean you can take someone to a GP if you want to but generally after hours, that's not possible. – *4, Paramedicine, Academic*

Are we there yet?

Multiple outcomes are necessary

I think you will need 30 measures? No, 10? No, I don't know how many. – *85, Paramedicine, Policymaker (National)*

I don't think there's one measure that just jumps out on its own. – *62, Parliamentarian*

Do they measure those sort of things for a GP? So if someone goes to a GP and the GP doesn't prescribe, do anything do they say, "Oh, why did the patient go to a GP? What is that for?". How well a GP is measured from a what happens to that patient through the journey? You know, was it the right decision to treat the patient in a certain way or not? – *51, Paramedicine, Clinician-Researcher*

We have to adhere to the triple aim, and over the top of the triple aim, which is obviously yeah, patient outcomes, patient experience, clinician experience and the cost, you know, to be able to have a cost-effective service. – *3, Chief Officer (State)*

How do you measure from an elderly faller to a 3 year old with a lac on the head, to someone who's got complex social needs? How do you measure that? – *51, Paramedicine, Clinician-Researcher*

Are we value adding to this event? We have to use a mix of operational and pre-hospital quality of outcome measures. Bring in all that other stuff around satisfaction, transportation rate, health improvement. One thing that's probably really important to zone in on is that whatever we're doing, we shouldn't be creating something new, we should be adding a module onto the existing outcome measures that are being developed. – *80, Paramedicine, Manager*

Removing that paternalistic medicine. – *51, Paramedicine, Clinician-Researcher*

There is really no standardized approach to reporting or evaluating outcomes in Australia. I think the days of using KPIs, you know, in isolation are no good. I think operational outcome measures is really important. But they need to be cross-referenced with clinical outcome measures. – *80, Paramedicine, Manager*

Appropriateness of care

Are you doing meaningful work? Is this contributing to a greater good? – *21, Paramedicine, Academic*

Just use my cellulitis example, a bean counter could say that's a condition that should easily be managed in the community, doesn't need an ambulance. Whereas, for that person, going to hospital would be based on their social determinants of health and the context that they exist in, would be absolutely the right answer. And we should facilitate that. And so I don't know how you draw that line. – *5, Chief Officer (State)*

Was the situation addressed? Was the necessary communication achieved, so that the patient could have better ongoing management without being required to call 000? – *30, Chief Officer (National)*

Palliative care, that might actually be an outcome that the person wanted is to die naturally at home. But it's captured in figures as possibly being the negative thing. – *51, Paramedicine, Clinician-Researcher*

Am I doing the right thing for this person? I don't think they're going to die. But is this actually the right thing for them?". So are they going down the correct healthcare pathway. So yeah, how do you measure that? – *51, Paramedicine, Clinician-Researcher*

We literally sit in front of that person and go. What do you need? And that's the driver. – *51, Paramedicine, Clinician-Researcher*

We've delivered an astounding health service, and I might not have given one medication. I might not have done any paramedic intervention, but purely listening to, assessing and providing advice, I've given that person good health care. And that needs capturing. – *51, Paramedicine, Clinician-Researcher*

Cost

I definitely think that the health economics is a really important and largely missing piece. – *26, Paramedicine, Executive*

Look. The ambulance service is all about cost benefit. – *68, Paramedicine, Academic*

We have to be cost responsible. Primary care is absolutely a cheaper model for the delivery of services. – *5, Chief Officer (State)*

The cost benefit analysis. We know that putting someone in a hospital bed is the single most expensive thing that you can do. So anything, really, nearly anything outside of putting someone in the hospital bed is going to probably show some sort of cost benefit. – *9, Paramedicine, Clinician*

It's all the cost, efficiency, and all. – *68, Paramedicine, Academic*

There's a cost behind this. We've got to be efficient. – *51, Paramedicine, Clinician-Researcher*

One of the problems is our managers and up through into sort of ministerial level scratching their head saying, 'What am I paying for here?' So value for money, I think, is really essential. – *1, Paramedicine, Manager*

Those measures are reasonably producible. They're easily understandable and produce a nice sort of you can get a graph and stick in front of an executive. This is cost-effective, and they can go back to the Health Minister and say, look, we're saving you lots of money. But again, I want to come back to the point that we're actually providing health care for an individual, so I think everything needs to be in context. – *51, Paramedicine, Clinician-Researcher*

I would suggest that when I'm reading the literature there's just not nearly enough information on health economics. At the end of the day for me it comes down to the buck, right? How much is it going to cost? How much is it going to save us? There is more and more of health economic evaluations coming through, which is great. But I would suggest that we need more information in that space. – *6, Paramedicine, Academic*

If it saves any health service money, then I think they're going to back it. – *14, Paramedicine, Academic*

Economic evaluations and strong economic evaluations to really show the value that these have. In my opinion, there needs to be a robust framework for economic evaluations of these models of care. – *95, Paramedicine, Academic*

Unfortunately, in health, money talks, doesn't it? So there has to be some sort of financial thing in the end. – *14, Paramedicine, Academic*

There's some issues with cost and the benefits there, because we need to know how many patients an

extended care paramedic might need to see, and so you've got to try to work out the balance of that. – *55, Medicine (Emergency) Policy & Clinician*

Financial data. I think it's important how much is this costing us. – *72, Paramedicine, Academic*

I'd be cautious. In this space, there's untapped, almost unlimited, demand. If you are there to provide a service in the space, then the expectations may rise – so rather than a reduction in triple 000 load you may see a huge spike in calls. – *55, Medicine (Emergency) Policy & Clinician*

When the national home doctor service was operating… after hours call outs were all billed under the national service, but costs were not sustainable to continue the service the way it was. – *55, Medicine (Emergency) Policy & Clinician*

Whether there is a significant saving or not. I think it's all money isn't it? I think it's all about the money and whether there was a saving. – *75, Medicine (ED), Policy (State)*

Economic outcomes - and, by the way, I wouldn't know the least bit of what I've talked about – but I would suggest that a lot of the time we have a bucket of money that we give the ambulance services access to, to do the things that ambulance services do. – *20, Paramedicine, Academic*

Are we actually saying a cost shifting from State funding to just the Federal primary care system. So is it actually even saving any money? – *95, Paramedicine, Academic*

Safety

I would want to know if there's any adverse outcome, those near misses. – *75, Medicine (ED), Policy (State)*

The other bit that we want to see is that longitudinal outcome for the patient. Did they die? Did they get sicker? Do we transfer them to hospital and then they got sicker? – *3, Chief Officer (State)*

You're looking at degrees of health outcome, although they can be really tricky, because even the way you measure health outcomes and adverse events and things like that, that's a hard space to get right. – *55, Medicine (Emergency) Policy & Clinician*

Adverse incidents would be interesting. What would be deemed an adverse event from this? I'm not sure. – *30, Chief Officer (National)*

Adverse events. I mean, realistically, if we've got patient's safety actually at the forefront, adverse events should be reduced if you've got the right people doing the right job. And adverse events are always going to happen but as long as they've been appropriately risk managed. – *48, Paramedicine, Clinician-Academic*

Adverse outcomes is important. I think transportation rates, but more importantly, call backs. If we go to someone and send a low acuity or an ECP and we get called back within 24-48 hours then they haven't done what they needed to do. – *95, Paramedicine, Academic*

Patient experience

It came down to patient experience. That to me is how that person has perceived their care. And I just want to emphasize that's different from satisfaction as well. I think it's sort of important for anyone looking at those measures, that they are two very different things. – *51, Paramedicine, Clinician-Researcher*

Someone goes to the doctor and antibiotics not necessarily the best thing. Do we give them antibiotics or do we educate them that they actually don't need antibiotics. They then walk away and go, "Well did I get what I wanted?". And that's why satisfaction is like a dangerous measure, because a GP could just go, "There you go. There's your antibiotics. Bye". That patient's satisfied. – *51, Paramedicine, Clinician-*

Researcher

Let's measure it on their level. So not only does it give you a picture of is this working, but it also gives you a guide on what's needed. – *51, Paramedicine, Clinician-Researcher*

Patient satisfaction is a thing. It's a little bit of a conundrum for me, because a happy patient does not necessarily equal excellent health care. But I think patient satisfaction would be important to measure. – *75, Medicine (ED), Policy (State)*

Patient satisfaction is a big marker. – *7, Paramedicine, Clinician*

Satisfaction from patients in relation to care given and health improvement. – *87, Nursing (Emergency), Clinician*

Patient satisfaction – but that would be likely to be high. – *55, Medicine (Emergency) Policy & Clinician*

Patient satisfaction and feedback are important. – *72, Paramedicine, Academic*

Because it's only one episode of care it's very hard. You're not going to see long term clinical gains one way or another. I think that I would be looking at PREMs and PROMs. – *66, Patient Representative*

Satisfaction is a really hard thing to measure. More and more we're seeing PROMS, you know, the patient recorded outcome measures. – *30, Chief Officer (National)*

Practitioner satisfaction

One outcome would be fatigue management for my regular crew. – *67, Paramedicine, Manager*

We want to bring joy to everyone, right, in their workplace? So is this good for paramedics? – *66, Patient Representative*

What paramedics themselves want. Is this the field that they actually wanted to enter into? I'm not sure… the career path of our paramedics is something that has to be brought into consideration. – *55, Medicine (Emergency) Policy & Clinician*

You're training a lot of people to be really skilled in their service, and you need to make sure that they are looked after, have the job satisfaction, and the support, so that in five years' time you're not trying to train another massive cohort of people. – *55, Medicine (Emergency) Policy & Clinician*

I was really happy to hear [other focus group participant] talk about the paramedic satisfaction, instead of the patient satisfaction, because I think that's the end of the day, if we actually do things to the detriment of our clinicians, then we won't have the workforce. – *14, Paramedicine, Academic*

Fatigue on crews. If we don't do something, if these other models of care aren't established, and that takes a whole heap of changes within Department of Health and Ambulance, then my concern is what's happening to the workforce. We want to attract and retain paramedics into the service, but you need to make sure the right support services are there and you're not fatiguing them with chronic workloads that have a negative effect on them… It's a hard job. It should be a rewarding job, but when you're going from one chronic patient to another, you need to make sure you're looking after your staff. Staff safety, staff welfare should be part of this conversation as well. – *45, Parliamentarian*

Gen Alpha is in employment now, they're going to shift every three to five years. Without question they're going to say "Okay. Done that for a little bit. Off to something new". That's what's coming so grab the opportunity. Let people move if they want to. We should just adapt. That's okay. – *5, Chief Officer*

(State)

We want to be very careful that we don't make the assumption that paramedics are going to be any more willing to work in these spaces than a nurse is. What concerns me a little bit is that when we do start opening up other areas of practice to paramedics, there might be this little bit of a surge initially into areas because it's new and it's giving paramedics opportunities that they haven't had before. But I think we're kidding ourselves if we think that we're going to suddenly find this whole percentage of the paramedic workforce that's gonna love to go and work in aged care, that's gonna love to go and work in remote practice. We're no different to nurses. We're all the same people, we all have the same issues as nurses do when it comes to wanting to work in these spaces. So I think we've got to be very careful that we don't try and sell paramedicine, as somehow a gap filler where nurses aren't going. – *9, Paramedicine, Clinician*

Satisfaction in the job by the paramedic. – *67, Paramedicine, Manager*

Reviews on paramedic's satisfaction should be included. – *87, Nursing (Emergency), Clinician*

Outcomes for the workers I think are high on my list. – *21, Paramedicine, Academic*

Workforce wellbeing and satisfaction. – *85, Paramedicine, Policymaker (National)*

Re-presentation rates

Re-presentations would be a good measure. – *25, Medicine (ED), Clinician*

Did people represent to emergency services or primary care within 24 48 hours, etc. Now again I found that a challenging target. I guess if you're trying to look at efficiencies and cost savings that can come in there, but it needs to also be balanced with other information. It's not really a standalone performance indicator. – *51, Paramedicine, Clinician-Researcher*

Good outcomes for me are reducing patient presentations. – *7, Paramedicine, Clinician*

That the patient subsequently has sought health care, either from a hospital or a GP for the same condition. That loop out. – *52, Paramedicine, Policy (National)*

Reduction in low acuity calls, that might be a way of going they're no longer calling us for something that they shouldn't be calling us for. – *85, Paramedicine, Policymaker (National)*

ED transportation rate by specialist paramedics

I think these things need to be measured in non-transport rates. – *51, Paramedicine, Clinician-Researcher*

[For Community Paramedics] our average ED avoid rate is about 80%. – *1, Paramedicine, Manager*

The thing that worries me is when models like this focus entirely on diversion from emergency department… when I see a lot of models with KPIs around diversion and around the money, things can get a bit skewed, because you divert people to nowhere. – *64, Medicine (ED), Policy (National)*

You can record transfers, and the reasons for transfers, and you look at the total numbers in that space. – *55, Medicine (Emergency) Policy & Clinician*

Hospital diversion being the key indicator is not particularly a useful indicator at all. – *21, Paramedicine,*

Academic

Miscellaneous

The nursing profession and paramedicine profession are very different. I feel like at times the nurse will walk in and go, 'Right, now, let's go through a list of reasons why I can't treat you today'… paramedics come with that can do attitude, like, 'I'm going to try and achieve… I'm not going to preach no lift policy or any crap like that'… that's a quality that really lends itself to out of hospital care. – *1, Paramedicine, Manager*

It's very existential. Do you need an ambulance to be a paramedic? – *1, Paramedicine, Manager*

[We need to be] learning from other countries that have done similar things. – *51, Paramedicine, Clinician-Researcher*

I think there's a need for a balance of skills within the paramedics. – *12, Patient*

Appendix II: Consumer recruitment advertisement

Improving Australia's 000 ambulance services: Seeking participants for a 30 minute research interview.

Last year, ambulances responded to nearly 4 million Australians.

Approximately 60% of these 000 ambulance calls weren't for 'emergencies' (like a heart attack or asthma attack) but instead for 'urgent' or 'primary' healthcare (like a blocked catheter or having the flu).

The University of Melbourne is hoping to understand why this is, what service Australians would like from their 000 paramedics, and how we can improve our 000 services.

If you have ever called 000 for an ambulance for something that may not have been a medical emergency (or, alternatively, something that you were unsure was an emergency), we would greatly appreciate the opportunity to talk to you.

Participants will take part in a 30 minute group interview with up to 4 other participants over Zoom. You will be provided with a $15 voucher as thanks for your time.

Appendix III: Semi-structured focus group (or interview) guide

General notes for the interviewer:

- Interviewees are to be provided with a summary of the interview questions a minimum of two days in advance, to allow them time to reflect prior to presenting their views.
- Confirm that Consent Forms for all participants have been completed prior to the interview.
- As participants join the interview, remind them that the interview is being recorded.
- Remind all participants that the interview is confidential, and that they have agreed to not share the identities, opinions, or comments of any other interviewee.
- At the commencement of the interview, ask participants to introduce themselves for the recording in a single sentence.
- For those with a relevant background, clarify that this research is exclusively on reactive models within a '000' service. Proactive models and non-JAS models are excluded.
- The scripted statements and questions are intended as guides only – some interviewees will have no healthcare knowledge, while others will be specialists, and adapting the statements and questions to the audience while preserving the core meaning is entirely appropriate.
- Do not interrupt interviewees, and when an interviewee finishes talking wait for at least 5 seconds before speaking to ensure they have no further comments to add.
- All questions are to be asked, even when a later question has been discussed during a previous response. This is to give interviewees the opportunity to expand on their answers.
- Questions can be asked out of order, if the conversation naturally flows that way.
- Give each participant the opportunity to comment on each question. If all participants have engaged in a general conversation, it is appropriate to instead ask 'Does anyone have any additional thoughts to share?'

To commence the interview, the following statement will be read (modify as appropriate for participants):

Thank you for agreeing to be part of a focus group/interview for this research; we greatly appreciate your time and insights.

This interview is semi-structured. This means that these questions exist as prompts, but that the interview can take the form of a conversation, and you may be asked different questions or to expand on comments you make. There are no right or wrong answers. We are not seeking a particular outcome. Our goal is simply to learn your views on the topic.

This study is looking at all the factors influencing policy decision-making relating to paramedics who work for a '000' ambulance service and who specialise urgent and primary healthcare. Primary healthcare refers to the kind of conditions that you may normally see a GP about. These interviews do not assume you have any prior knowledge, and many participants have no knowledge of the emergency ambulance system. The goal of this research is ***not*** *to determine what the right or wrong course of action for these paramedic models is, but instead to understand all the different viewpoints on them.*

I will now provide you with some information about Australia's 000 ambulance services. Last year the Australian 000 ambulance services had a budget of $4.8 billion and responded to 3.9 million patients. Approximately 60% of these patients were not classified as emergency patients. Although this data is not exact, it does suggest that a large amount of '000' ambulance calls are not what the medical system would consider an emergency, and that these make up a significant expense.

Last year, Australian 000 ambulance services transported 85% of patients to an Emergency Department. Previous research has shown that with paramedic training it is possible to reduce this transportation rate by between 20% and 70%.

Every state and territory either has, is piloting, or is developing, paramedics specialising in urgent-primary care. These paramedics generally do a Master's degree, work alone in a non-ambulance vehicle, and have extra skills and medications. However, they are not widely used or available in most locations, and more research is needed to determine how to use them most effectively.

Questions:

1. What are your opinions on paramedics specialising in urgent-primary care?

2. From your perspective would make these models work well or not work?

3. What outcomes do you believe are most important to measure in these models? (E.g. adverse events, health improvement, satisfaction, transportation rate, cost, etc.)

4. Do you have any final thoughts you would like to share?

To conclude the interview, complete the following statement and steps:

Thank you.

That concludes the interview. I will now stop the recording and explain the next steps.

From here, the interview will be transcribed. This transcript is considered your data, and you are entitled to a copy of the transcript. It may take several weeks before transcripts are available. Please note that other participants' comments will be redacted; the transcript would be of your own comments only. You are also entitled to edit this transcript and send it back to us – if you do this, we will use the edited transcript. Would you like a copy of the transcript?

All feedback will be de-identified and described only using a general descriptor of your role (e.g. 'a primary care specialist paramedic'). Transcripts will then undergo a process called thematic analysis. We may want to use quotes by some interviewees to describe a point – if we would like to use one of your quotes, you will separately be contacted and asked for permission to use your quote. This will include the verbatim quote and the context in which it is to be used. You are free to refuse this without it affecting your participation in the study in any other way. You are also entitled to a copy of any academic articles published out of this research. It may take over a year before articles are available. Would you like a copy of any academic articles?

Thank you again for your assistance with this research.
